# Peptide dendrimers transfecting CRISPR/Cas9 plasmid DNA: optimization and mechanism†

**DOI:** 10.1039/d4cb00116h

**Published:** 2024-07-29

**Authors:** Susanna Zamolo, Elena Zakharova, Lise Boursinhac, Florian Hollfelder, Tamis Darbre, Jean-Louis Reymond

**Affiliations:** a Department of Chemistry, Biochemistry and Pharmaceutical Sciences, University of Bern Freiestrasse 3 3012 Bern Switzerland tamis.darbre@unibe.ch jean-louis.reymond@unibe.ch; b Department of Biochemistry, University of Cambridge 80 Tennis Court Road Cambridge CB2 1GA UK

## Abstract

Gene editing by CRISPR/Cas9 offers great therapeutic opportunities but requires delivering large plasmid DNA (pDNA) into cells, a task for which transfection reagents are better suited than viral vectors. Here we performed a structure–activity relationship study of Z22, a d-enantiomeric, arginine containing, lipidated peptide dendrimer developed for pDNA transfection of a CRISPR/Cas9 plasmid co-expressing GFP. While all dendrimer analogs tested bound pDNA strongly and internalized their cargo into cells, d-chirality proved essential for transfection by avoiding proteolysis of the dendrimer structure required for endosome escape and possibly crossing of the nuclear envelope. Furthermore, a cysteine residue at the core of Z22 proved non-essential and was removed to yield the more active analog Z34. This dendrimer shows >83% GFP transfection efficiency in HEK cells with no detrimental effect on cell viability and promotes functional CRISPR/Cas9 mediated gene editing. It is accessible by solid-phase peptide synthesis and therefore attractive for further development.

## Abstract 2

We optimize a peptide dendrimer transfection reagent to deliver a large CRISPR/Cas9 plasmid DNA into cells, elucidate its delivery mechanism, and demonstrate its gene editing efficiency.

## Graphical abstract

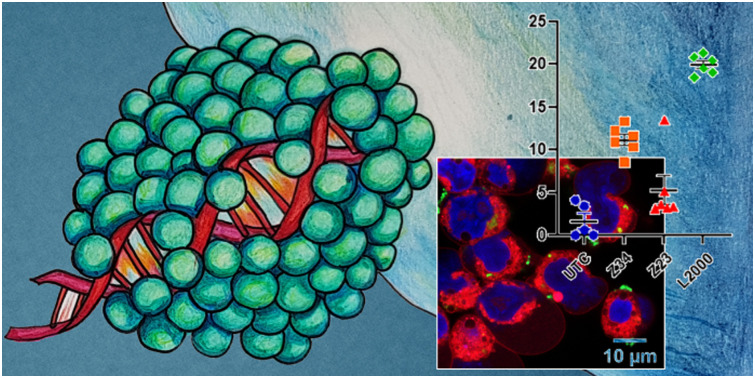

## Introduction

CRISPR/Cas9 and related gene editing tools have transformed the practice of biology and offer promising opportunities for therapy.^1^ One critical challenge for this technology remains the delivery into cells of the relatively large 9 kb plasmid DNA (pDNA) required for gene editing, which is beyond the encapsulation capacity of typical viral vectors (4.7 kb for adeno-associated virus), implying that non-viral vectors such as polycations offer promising alternatives.^2–5^ Among the various nanoscale systems developed for nucleic acids delivery,^6–8^ dendrimers of diverse architectures have been extensively studied as DNA and RNA transfection reagents.^9,10^ Extending on our interest in developing peptide dendrimers as transfection reagents for nucleic acids,^11–15^ we recently reported the arginine containing d-enantiomeric lipidated peptide dendrimer Z22 as transfection reagent for delivering a CRISPR/Cas9 pDNA into cells and tissue models with higher efficiency and lower cell toxicity compared to the reference reagent lipofectamine 2000 (L2000).^16^

Dendrimer Z22 was obtained by modifying G123KL, a previously reported dendrimer requiring addition of lipofectin for transfection of plasmid DNA,^11,12,14^ but which showed no activity when used alone. Our approach was similar to our previous design of the siRNA transfection dendrimer DMH13,^17,18^ and consisted in attaching a lipid chain to the dendrimer core to obtain a single component transfection reagent resembling poly(amidoamine) (PAMAM) transfection dendrimers.^9^ Compared to PAMAM dendrimers which are difficult to synthesize and purify, the advantage peptide dendrimers is that they can be obtained in pure form by standard solid-phase peptide synthesis, which also gives easy access to dendrimer libraries for property optimization. In the optimization leading to the single component pDNA transfection dendrimer Z22, we extended the core of G123KL with one or two side-chain palmitoylated or stearoylated lysines or a penta-leucine (Z1–Z4) and added a cysteine (Z12) or alanine (Z15) residue, which enabled detectable transfection of the 9kb CRISPR/Cas9 plasmid in HEK and HeLa cells (Table 1). Further and more significant transfection activity increases were achieved by switching to d-enantiomeric residues (Z20) and exchanging lysines to arginines, leading to dendrimer Z22 surpassing the reference reagent L2000 in transfection efficiency. Mechanistic studies showed that Z22 aggregates and binds pDNA at pH 7.4 to form nanoparticles that enter cells by endocytosis. Endosome acidification leads to protonation of the eight amino termini of the dendrimer inducing disaggregation and leading to endosome escape and delivery of the pDNA cargo into the cells,^16^ a mechanism similar to that of siRNA transfection dendrimer DMH13.^17,18^

**Table 1:** Transfection efficiency and binding properties of the peptide dendrimers

No.	Sequence^a^	% GFP pos. HEK cells^b^	% viability HEK cells	% GFP pos. HeLa cells^b^	% viability HeLa cells
L2000	—	50.7 ± 14.6	89 ± 1	30.2 ± 12.6	92 ± 7
G123KL	(KL)_8_(*K*KL)_4_(*K*KL)_2_*K*GSC	8.0 ± 2.7	52 ± 9	4.0 ± 1.9	n.d.
DMH13	(kl)_8_(*k*kl)_4_(*k*ll)_2_*k*k(C_16_)k(C_16_)	28.9 ± 2.1	96 ± 6	2.6 ± 2.4	n.d.

Initial optimization (previous work):					
Z1	(KL)_8_(*K*KL)_4_(*K*KL)_2_*K*K(C_18_)	10.7 ± 3.2	64 ± 7	2.4 ± 2.2	51 ± 6
Z2	(KL)_8_(*K*KL)_4_(*K*KL)_2_*K*K(C_18_)K(C_18_)	11.7 ± 3.6	79 ± 11	2.0 ± 1	47 ± 3
Z3	(KL)_8_(*K*KL)_4_(*K*KL)_2_*K*LLLLL	7.2 ± 2.8	76 ± 7	3.8 ± 2.5	51 ± 5
Z4	(KL)_8_(*K*KL)_4_(*K*KL)_2_*K*K(C_16_)	7.1 ± 1.8	89 ± 11	1.9 ± 0.4	88 ± 11
Z12	(KL)_8_(*K*KL)_4_(*K*KL)_2_*K*K(C_18_)C	12.0 ± 3.0	80 ± 5	3.8 ± 2.5	112 ± 10
Z15	(KL)_8_(*K*KL)_4_(*K*KL)_2_*K*K(C_18_)A	10.1 ± 2.4	71 ± 7	1.0 ± 0.9	107 ± 10
Z20	(kl)_8_(*k*kl)_4_(*k*kl)_2_*k*k(C_18_)c	51.6 ± 12.5	85 ± 9	34.2 ± 13.1	85 ± 9
Z22	(rl)_8_(*k*rl)_4_(*k*rl)_2_*k*k(C_18_)c	63.6 ± 3.8	64 ± 4	49.9 ± 8.5	92 ± 7

Arginines, l-enantiomers:					
Z23	(RL)_8_(*K*RL)_4_(*K*RL)_2_*K*K(C_18_)	29.1 ± 4.8	83 ± 2	2.9 ± 0.8	95 ± 5
Z24	(RL)_8_(*K*RL)_4_(*K*RL)_2_*K*K(C_18_)C	35.5 ± 5.5	86 ± 1	3.3 ± 0.6	91 ± 6
Z25	(RL)_8_(*K*RL)_4_(*K*RL)_2_*K*K(C_18_)A	30.7 ± 6.0	85 ± 2	4 ± 0.7	104 ± 8

Lysines, d-enantiomers:					
Z26	(kl)_8_(*k*kl)_4_(*k*kl)_2_*k*k(C_18_)	56.8 ± 9.8	91.3 ± 0.2	15.8 ± 5.1	91 ± 5
Z27	(kl)_8_(*k*kl)_4_(*k*kl)_4_*k*k(C_18_)k(C_18_)	28.3 ± 9.5	74.8 ± 0.7	11 ± 2.6	94 ± 5
Z28	(kl)_8_(*k*kl)_4_(*k*kl)_2_*k*lllll	16 ± 6.3	103 ± 0.2	5.7 ± 1.7	101 ± 7
Z29	(kl)_8_(*k*kl)_4_(*k*kl)_2_*k*k(C_16_)	52 ± 11.0	87 ± 1	16.4 ± 5.9	95 ± 5
Z30	(kl)_8_(*k*kl)_4_(*k*kl)_2_*k*k(C_18_)a	53.8 ± 10.5	75 ± 1	20.3 ± 5.0	91 ± 8
Z31	(kl)_8_(*k*kl)_4_(*k*ll)_2_*k*k(C_18_)	22.8 ± 3.6	96 ± 2	11.5 ± 2.4	91 ± 6
Z32	(kl)_8_(*k*ll)_4_(*k*ll)_2_*k*k(C_18_)	1.4 ± 1.1	111 ± 1	1.4 ± 0.8	97 ± 4

Arginines, d-enantiomers:					
Z33	(rl)_8_(*k*rl)_4_(*k*rl)_2_*k*k(C_16_)	80.7 ± 7.2	84 ± 1	30.5 ± 12.2	93 ± 6
Z34	(rl)_8_(*k*rl)_4_(*k*rl)_2_*k*k(C_18_)	83.4 ± 5.3	101 ± 4	41.2 ± 3.9	94 ± 6
Z35	(rl)_8_(*k*rl)_4_(*k*rl)_2_*k*k(C_18_)a	72.4 ± 5.0	72 ± 5	52.1 ± 3.3	94 ± 6
Z36	(rl)_8_(*k*rl)_4_(*k*ll)_2_*k*k(C_18_)	35.6 ± 2.1	87 ± 2	6.9 ± 1.2	92 ± 6
Z37	(rl)_8_(*k*ll)_4_(*k*ll)_2_*k*k(C_18_)	1.8 ± 2.8	103 ± 1	0.9 ± 0.3	95 ± 5

a One-letter code amino acids are used, with upper case letters referring to l-amino acids and lower case letters to d-amino acids. The branching lysine residue are written in italics, C-termini are carboxamide CONH_2_, and all N-termini are free. C_16_/C_18_ are palmitoyl/stearoyl groups at the lysine side chain. Full structural formula and SMILES are provided in the ESI.

b Transfection efficiency in HEK293 and HeLa cells measured as percentage of GFP positive cells after 48 h transfection. Transfection conditions: cells are transfected with pDNA coding for CRISPR-Cas9/GFP. Peptide dendrimers/pDNA complexes are formed at N/P 5 (175–240 pmol (1.75–2.4 μM) of peptide dendrimers and 250 ng (0.42 nM) of pDNA in 100 μL of OptiMEM per well in 96 well plate). L2000 (2 : 1, v/w, L2000 : pDNA) is used as positive control. Transfection efficiency is detected after 48 h incubation by FACS and expressed in percentage of transfected cells relative to the whole cell population (1 × 10^4^ events).

**Fig. 1:**
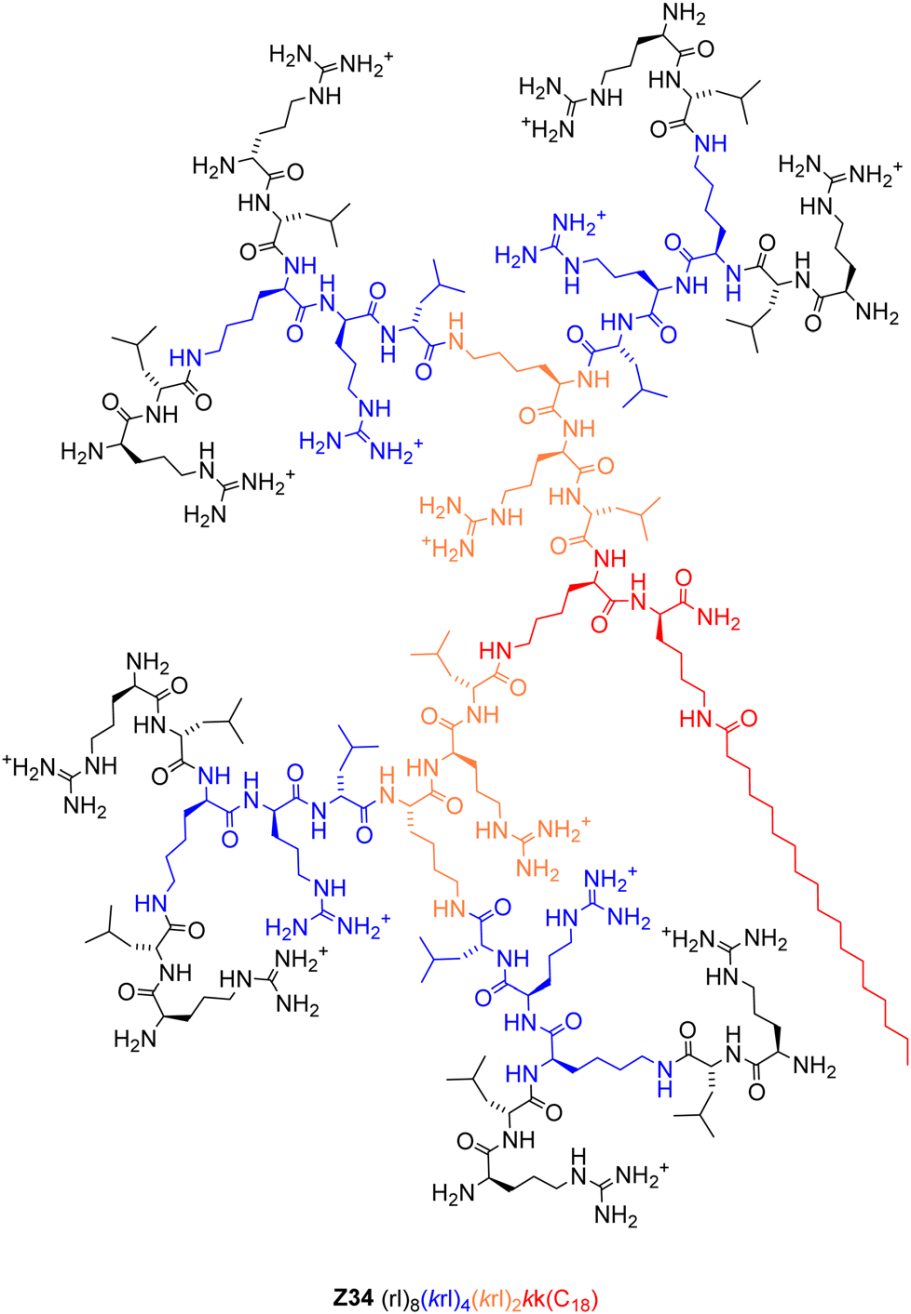
Structural formula of the optimized pDNA transfection dendrimer Z34.

Herein we report a structure–activity relationship (SAR) study on the transfection activity of Z22. By repeating the dendrimer optimization in both enantiomeric series, we confirm that the exchange of lysines to arginines as cationic residues and the switch do d-enantiomeric residues independently contribute to pDNA transfection efficiency of this dendrimer. Furthermore, we show that the presence of a cysteine residue at the core of dendrimer Z22, taken from the original design of G123KL, is not necessary and even decreases transfection efficiency, leading to the identification of the simpler but more active transfection dendrimer Z34 (Fig. 1). In terms of the transfection mechanism, we show that all dendrimers investigated bind pDNA and internalize their cargo into cells, and that the inactivity of l-enantiomers results from their proteolytic degradation since their transfection activity can be rescued in the presence of protease inhibitors. This effect highlights the role of dendrimer structure in triggering endosome escape, and possibly in permeabilizing the nuclear envelope, which are required after internalization for functional expression of the plasmid. Finally, we show that our transfection system delivers a functional gene editing CRISPR/Cas9.

## Results

### Structure–activity relationship study of transfection dendrimer Z22

To better understand the activity of our pDNA transfection dendrimer Z22, we designed analogs Z23–Z37 inspired from the original optimization series Z1–Z22. All dendrimers were prepared by Fmoc solid-phase peptide synthesis and obtained as pure products from preparative reverse-phase HPLC (Table S1, ESI†). All dendrimers were evaluated for their transfection activity on HEK cells, which are particularly prone to transfection,^7^ as well as on HeLa cells. We used a CRISPR/Cas9 pDNA co-expressing a GFP using an optimized N/P ratio of 5, counting GFP positive cells by flow cytometry. While cell viability was only weakly affected by the dendrimers, their transfection activity varied in function of dendrimer amino acid sequence and chirality (Table 1).

### Transfection increases with arginines and d-chirality

We first investigated Z23–Z25 as the arginine analogs of the initial l-enantiomeric dendrimers Z1, Z12 and Z15 to see if the pDNA transfection activity increase observed between the lysine containing d-enantiomer Z20 and its arginine analog Z22 was broadly valid. Indeed, pDNA transfection activity on HEK cells raised from 10–12% transfected cells using the lysine containing dendrimers Z1, Z12 and Z15, to 29–36% transfected cells with the arginine containing analogs Z23–Z25, confirming the effect of this residue substitution on transfection efficiency. However, the Lys → Arg exchange was insufficient to obtain significant transfection of HeLa cells.

To probe the role of dendrimer chirality in transfection activity independently of Lys → Arg mutations, we next prepared analogs of the weakly active l-enantiomeric lysine-containing hydrophobic core variants Z1–Z4 and Z15 in their d-enantiomeric form (Z26–Z30) expected to be more active. Transfection assays indeed showed that these d-enantiomeric lysine containing dendrimers exhibited much better levels of transfection on HEK cells (16–57%) and showed some activity on HeLa cells (6–20%) compared to their l-enantiomers (7–12% on HEK cells, <4% on HeLa cells). Note that the original sequence Z20 (52% on HEK cells, 34% on HeLa cells) remained the most active dendrimer in this d-enantiomeric lysine series, on par with L2000 (51% and 30%).

### Transfection is unaffected by cysteine

In the l-enantiomeric arginine series (Z23–Z25) which only transfected HEK cells significantly, the cysteine containing dendrimer Z24 was slightly more active than its analogs without cysteine (Z23) or with alanine (Z25). The same trend occurred in the d-enantiomeric lysine series for activity on HeLa cells (Z20 > Z26 and Z30), however these three dendrimers had comparable activities on HEK cells, with Z26 lacking a cysteine residue being slightly more active (57%) and overall best in the lysine series dendrimers.

In view of these unpredictable effects of slight core modification on transfection activity, we prepared analogs of our previous best transfection dendrimer Z22, which was d-enantiomeric and arginine containing, by removing the cysteine residue and placing a side chain palmitoylated (Z33) or stearoylated (Z34) lysine at the core, or by replacing cysteine by alanine (Z35). Removal of cysteine increased transfection efficiency, with an increase for the simple cysteine deletion mutant Z34 (83% on HEK cells, 41% on HeLa cells) and the Cys → Ala mutant Z35 (72% for HEK cells, 52% on HeLa cells), suggesting that transfection did not depend on a cysteine specific effect. Furthermore, Z34 bearing a C_18_ core lipid remained slightly more effective for transfection than Z33 with a slightly shorter C_16_ lipid, in line with our previously reported observations that analogs of Z1 with shorter lipid chains (C_8_, C_10_, C_12_) did not transfect pDNA (0.1–3.1% GFP positive HEK cells).^16^

The lack of a positive cysteine effect was further evidenced by the fact that transfection was unaffected in the presence of dithiothreitol (DTT) or Ellman's reagent (DTNB), which act as activator or inhibitor of protein disulfide isomerases (PDI) present on the cellular surface (Fig. S1, ESI†). These results indicated that the thiol group in the cysteine side chain did not improve transfection, in contrast to many other synthetic carriers functionalized with thiols or strained disulfides which interact with PDI and related disulfide exchange membrane proteins and thereby undergo facilitated cellular uptake.^19–22^

### The arg–leu dipeptide branches of Z34 are optimal for pDNA transfection

The repeated amphiphilic dipeptide across the G1, G2 and G3 branches of Z22 (arg–leu) was borrowed from the original G123KL co-transfection dendrimer (Lys–Leu), for which exchanges of the G1 and G2 branches to Leu–Leu dipeptides decreased DNA co-transfection with lipofectin.^11^ On the other hand optimization of siRNA transfection had resulted in dendrimers such as DMH13 containing a leu–leu dipeptide in the G1 branches and lys–leu dipeptides in G2 and G3.^12,17^

Since doubling of the side-chain stearoylated lysine core of Z26 to form Z27 only led to a slight decrease in pDNA transfection, we tested whether lys → leu mutations in the G1 and G2 branches of Z26, which would result in a less drastic change in charge/hydrophobicity ratio, might increase its pDNA transfection efficiency. However, this was clearly not the case. Indeed, lys → leu mutations in the G1 (Z31) and G1 + G2 branches (Z32) of Z26 led to a progressive loss of activity. The same effect occurred when modifying the d-enantiomeric arginine dendrimer Z34 to form Z36 (arg → leu in G1) and the entirely inactive dendrimer Z37 (arg → leu in G1 and G2), confirming the optimal sequence of the repeated dipeptide dendrimer branches for pDNA transfection by our dendrimers (lys–leu in Z26 and arg–leu in Z34).

### All dendrimers bind pDNA and internalize their cargo into cells

**Fig. 2:**
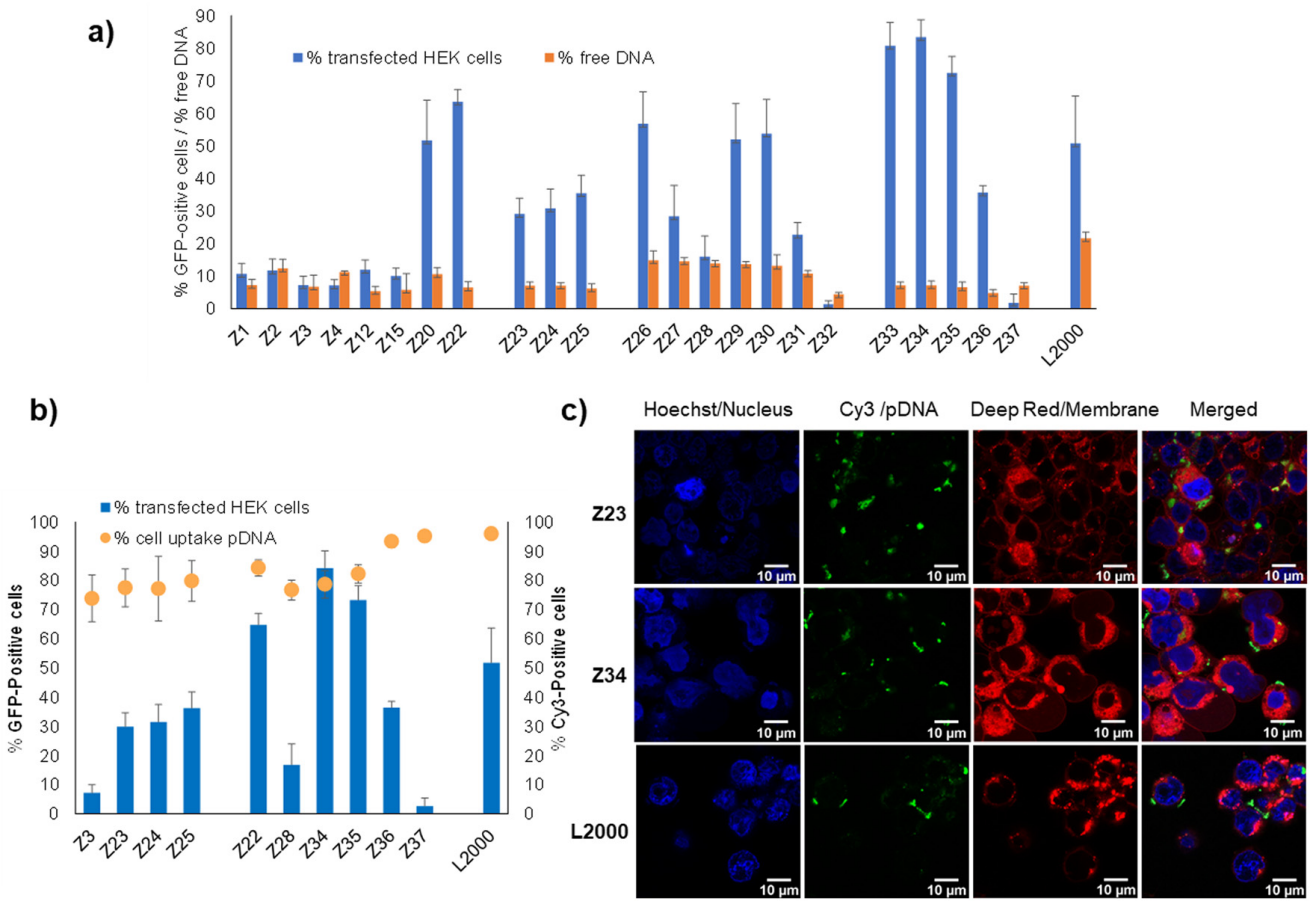
pDNA binding and cellular uptake by transfection dendrimers. (a) Percentage of transfected HEK cells (see Table 1 for conditions) and percentage of free DNA as measured by fluorescence from intercalation in pDNA/peptide dendrimer complex at N/P 5 (200 μL, final concentration 0.085 nM of pDNA and 0.35–0.48 μM of peptide dendrimer or 1 μg mL^−1^ of L2000) by Quant-It PicoGreen and normalized to the value of CRISPR-Cas9/GFP pDNA alone. (b) Comparison between peptide dendrimers transfection efficiency and cellular internalization on HEK293 cells. Cells are transfected with CRISPR-Cas9/GFP pDNA or Cy-3 conjugated CRISPR-Cas9/GFP pDNA. Transfection efficiency and internalization capacity are detected by FACS after 48 h and expressed in percentage of transfected cells relative to the whole cell population (1 × 10^4^ events). (c) Confocal microscopy images of HEK293 cells, incubated for 4 h with pDNA complexes of peptide dendrimers and L2000, under transfection conditions previously described. Three channels were acquired sequentially: Hoechst for nuclei staining in blue, Cy-3 labelled plasmid DNA in green, cellular membrane in red. Images are recorded on a Leica SP8 confocal microscope with lens x60. Scale bar 10 μm.

Binding to pDNA by the dendrimers, measured by the percentage of unbound pDNA at N/P ratio of 5 quantified using the PicoGreen assay, was much stronger for all dendrimers in our study (<15% free DNA) compared to L2000 or G123KL, which only weakly bound pDNA (22–35% free DNA). pDNA binding by the dendrimers was unrelated to their transfection efficiency. For example, the weakly active l-enantiomer Z1 (11% transfection of HEK cells, 7% free DNA) bound pDNA more strongly than its active d-enantiomer Z26 (57% transfection of HEK cells, 15% free DNA), while Z23 (29% transfection of HEK cells) and its more active enantiomer Z34 (83% transfection of HEK cells) bound pDNA to the same extent (7% free DNA). Note that the entirely inactive dendrimer Z32 (1.4% transfection of HEK cells) bound pDNA most strongly (4.4% free DNA) (Fig. 2(a)).

Furthermore, measuring cellular uptake of a fluorescence-labeled pDNA by flow cytometry on a selection of our dendrimers showed that the dendrimer–pDNA complexes were internalized into cells independent of their overall transfection activity. For instance, over 80% of cells exposed to Cy3-labeled pDNA complexed with the l-enantiomeric lysine dendrimer Z3 with a pentaleucine core or its d-enantiomer Z28, both inactive in transfection, were fluorescence labeled. These levels were comparable to those achieved with l-enantiomeric arginine containing Z23–Z25 showing intermediate levels of transfection, as well as with the most active d-enantiomers Z22, Z34 and Z35, and their more hydrophobic inactive analogs Z36 and Z37 (Fig. 2(b)). The cellular uptake of the Cy3-labeled as visualized by confocal microscopy in the case of Z23 and Z34 was comparable to that achieved with L2000 (Fig. 2(c)).

### Endosome acidification is required for transfection

**Fig. 3:**
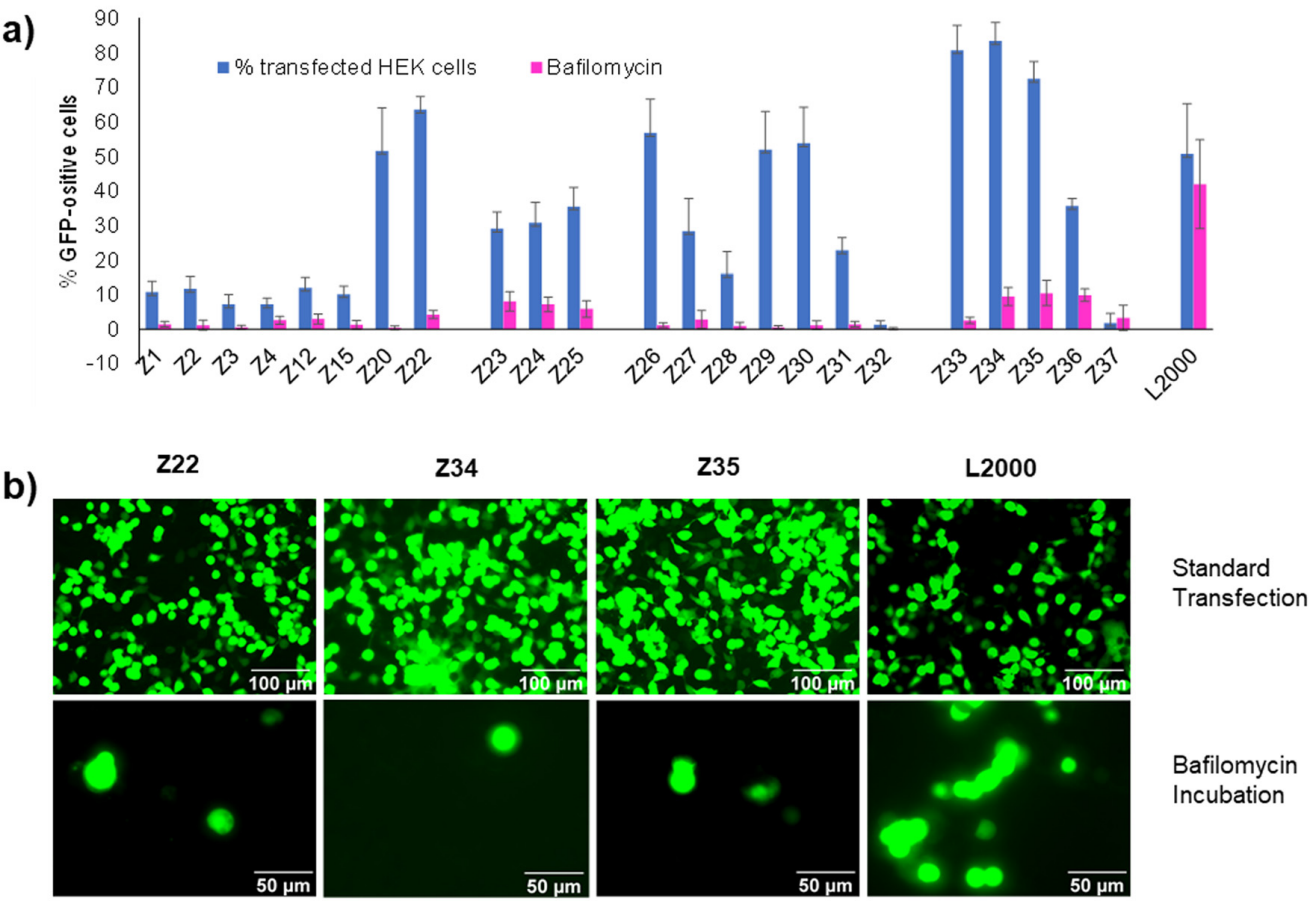
(a) Comparison between peptide dendrimers transfection efficiency on HEK293 cells without any treatment (in blue) and with Bafilomycin A1 incubation (in violet). See Table 1 and ESI† for details. (b) Fluorescence microscope images of HEK293 cells transfected under standard conditions or in the presence of Bafilomycin A1. Pictures taken by Nikon Eclipse TS100 (20×–40× objective) 48 h after transfection. Scale bar 100 μm and 50 μm.

We had shown previously that treatment with bafilomycin A1, a natural product inhibitor of V-ATPase which blocks endosome acidification,^23^ entirely inhibited pDNA transfection by our dendrimer Z22.^16^ The same effect was observed with our siRNA transfection dendrimer DMH13 and attributed to the protonation of the dendrimer N-termini, which are unusually acidic (p*K*_a_ ∼ 6.5), as a necessary step to enable endosomal membrane perturbation by the dendrimers.^17^ Indeed, inhibiting endosome acidification with bafilomycin A1 blocked pDNA transfection across the entire series of peptide dendrimers Z1–Z37 including weakly active ones, pointing to a common mechanism of cellular uptake (Fig. 3(a) and (b)). This effect is consistent with the strong pDNA binding observed across all dendrimers and the fact that all dendrimers carry eight N-termini sharing a similar chemical environment leading to their protonation below pH 6.5. Note that transfection by L2000 was unaffected by bafilomycin A1, in line with the proposed membrane fusion mechanism for the liposomes, a process less dependent on the pH of the endosomes and different from the escape mechanism of complexes with peptide dendrimers.^24^

### l-Enantiomeric dendrimers are degraded by proteolysis

**Fig. 4:**
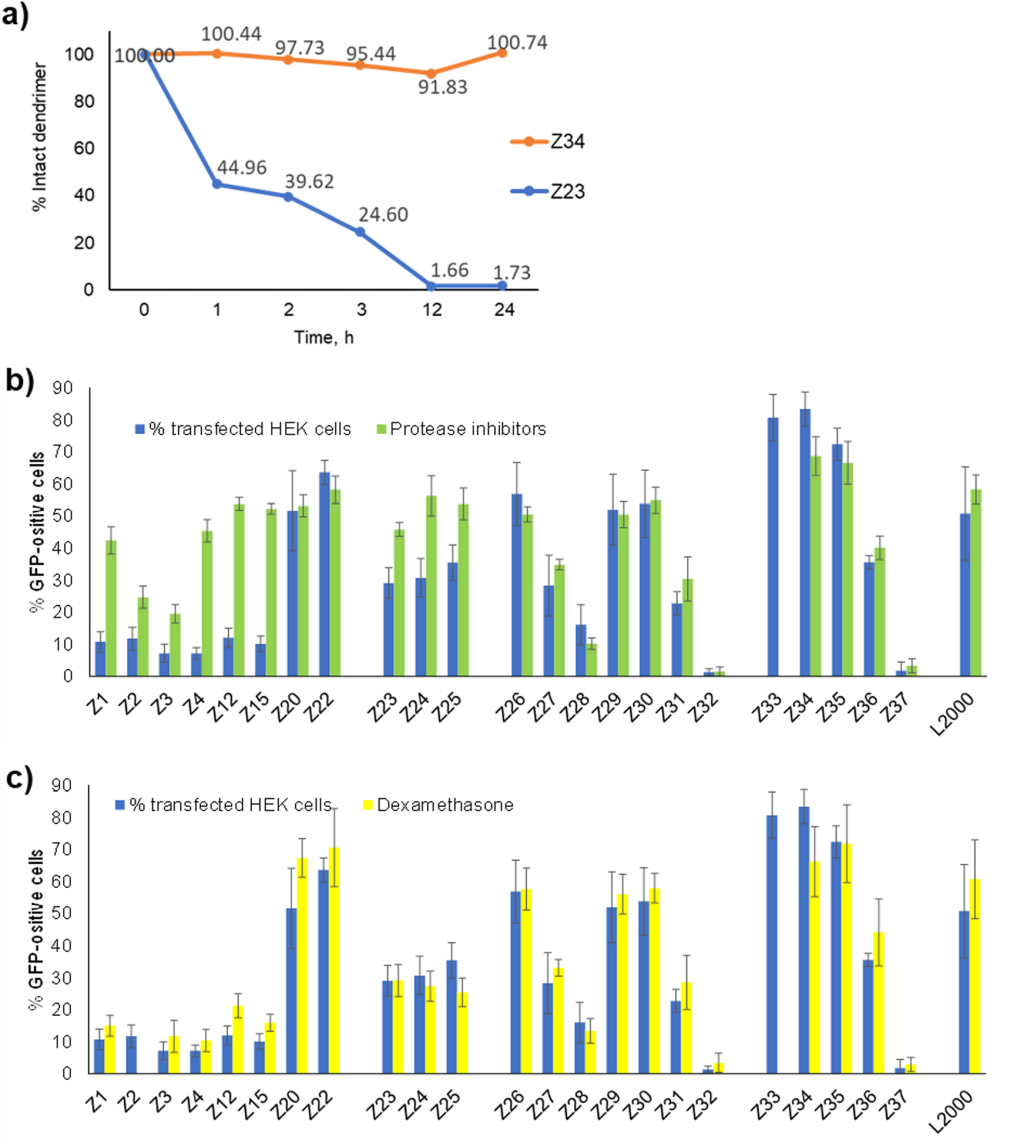
Effect of proteases and nuclear pore dilation on pDNA transfection. (a) degradation of l-enantiomeric dendrimer Z23 and its d-enantiomer Z34 by proteinase K. (b) Transfection of HEK cells without (blue) or with (green) protease inhibition cocktail (1:200 dilution) – added 1 h before transfection and 48 h after cell incubation with peptide dendrimers/pDNA complexes. (c) Comparison between peptide dendrimers transfection efficiency on HEK293 cells without any treatment (in blue) and with dexamethasone incubation (in yellow) (5 μM – added 1 h before transfection and 48 h after cell incubation with peptide dendrimers/pDNA complexes).

In view of the similar pDNA binding observed with both l-enantiomeric and d-enantiomeric dendrimers, the much stronger pDNA transfection activity of d-enantiomers over their l-enantiomers, such as Z26 (d-) *vs.*Z1 (l-) (57% *vs.* 11% transfected HEK cells) and Z34 (d-) *vs.*Z23 (l-) (83% *vs.* 29% HEK cells), must reflect a biological effect, presumably a degradation of l-enantiomeric dendrimers by proteases. Indeed, we have shown previously that l-enantiomeric peptide dendrimers are only moderately resistant to proteolytic degradation despite of the multi-branched topology of their peptide chain.^25^ Here we observed complete degradation of the l-enantiomer Z23 upon incubation with proteinase K over 12 h, while the d-enantiomer Z34 was undegraded even after 24 h under the same conditions (Fig. 4(a) and Fig. S2, ESI†).

To check if protease inhibition might affect pDNA transfection by our dendrimers, we performed transfection experiments in the presence or absence of a commercial protease inhibition cocktail containing serine, cysteine and aspartic protease as well as aminopeptidase inhibitors. Indeed, the activity of l-enantiomeric dendrimers increased strongly in the presence of the protease inhibition cocktail, in each case raising close to levels of the corresponding d-enantiomer (Fig. 4(b)). By contrast, the transfection activity of d-enantiomeric dendrimers was constant or even slightly decreased upon protease inhibition. For example, transfection by the l-enantiomeric lysine dendrimer Z1 increased from 11% to 42% with protease inhibition, close to the level of its d-enantiomer Z26 at 57% without and 51% with protease inhibition. A similar increase occurred with the l-enantiomeric arginine dendrimer Z23 (29% without → 46% with protease inhibition) compared to its d-enantiomer Z34 (83% without and 69% with protease inhibition).

### Transfection is not limited by crossing the nuclear envelope

The observation that protease degradation was a limiting factor in transfection by l-enantiomeric dendrimers implies that the full dendrimer structure was required for transfection, mostly likely to induce endosome escape by destabilizing the endosomal membrane. Nevertheless, successful pDNA transfection as detected here by the expression of GFP requires in addition crossing of the nuclear envelope to reach the nucleus where pDNA can be transcribed into a functional mRNA. The limiting role of the nuclear envelope in transfection of large CRISPR/Cas9 plasmid by polycations has been demonstrated by showing enhanced transfection in the presence of dexamethasone, a glucocorticoid which has been shown to dilatate the nuclear pore complex, which is the main gate of entry into the nucleus for large macromolecules.^26–30^

Here we found that transfection efficiency by our dendrimers was unaffected by the addition of dexamethasone, suggesting that nuclear entry was not a limiting factor (Fig. 4(c)). This effect might indicate that this step is not limiting in the HEK cells investigated here, or that our dendrimers also assist crossing of the nuclear envelope by permeabilizing its membrane, an effect which would result from their membrane destabilizing activity.

### Peptide dendrimers mediate CRISPR/Cas9 gene editing

Since our activity assay was based on detecting the fluorescence of GFP co-expressed with the CRISPR/Cas9 system, we finally also checked if the expressed CRIPR/Cas9 system was indeed functional for gene editing following dendrimer mediated transfection. We measured the efficiency of the CRISPR/Cas9 mediated genomic DNA modification using our best performing dendrimer Z34 and its l-enantiomer Z23 in comparison with L2000 and untreated controls (UTC).

**Fig. 5:**
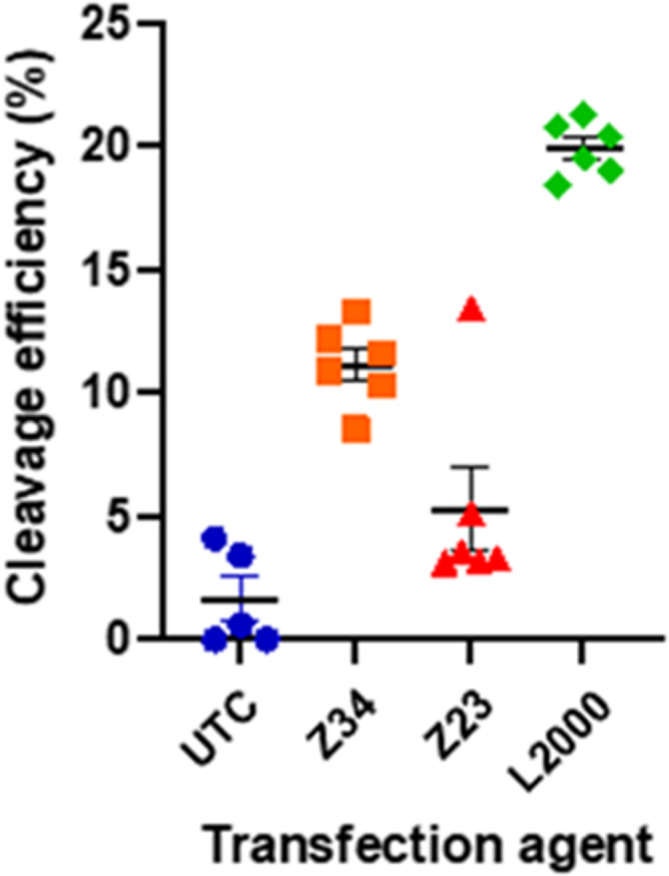
Cleavage efficiency of SpCas9 with a gRNA against the GUSB gene, 48 h after transfection in HEK293 cells. The total efficiency was measured by amplification of the region of the cut site, Sanger sequencing from the forward and reverse primer, and analysis with TIDE.

When transfected with and appropriate guide RNA (gRNA), SpCas9 will create a double stranded break in the genomic DNA complementary to the gRNA sequence used. The cell's DNA repair mechanism will then repair this break, often with mistakes causing insertion or deletions (indels) of a small number of nucleotides. A quantitative measure of the indels at the cut site, compared to a non-treated control, can thus indicate the efficiency of SpCas9 cleavage. This can easily be done using tracking of indels by decomposition (TIDE).^31,32^ The pDNA used here contained a cassette for expression of a single guide RNA (sgRNA) inducing cuts in the GUSB gene encoding for human β-glucoronidase. The cut efficiency was below 5% for UTC as well as for the Z23 mediated transfection, in line with the observed low transfection efficiency of this dendrimer caused by its proteolytic instability. By contrast, the Z34 mediated transfection resulted in a cut efficiency of 9–12% (Fig. 5 and Table S2, Fig. S3, ESI†). Although clearly lower that the 21% efficiency induced by L2000, this data confirmed the ability of our dendrimer to function as a CRISPR/Cas9 transfection reagent.

## Discussion

Here we extended our investigation of peptide dendrimers for transfection focusing on the case of large CRISPR/Cas9 plasmids for which non-viral vectors are promising to overcome limitations of viral delivery vectors. In our study of transfection dendrimers, we initially reported peptide dendrimer G123KL, as a reagent for DNA delivery in co-application with lipofectin, and showed that three successive generations of a lys–leu dipeptide connected *via* lysine branching residues were necessary for activity.^11^ Re-optimization of G123KL for siRNA delivery led to a slightly more hydrophobic dendrimer containing a leu–leu dipeptide at G1, again as co-transfection reagent with lipofectin.^12^ It should be mentioned that this dendrimer architecture, consisting of three generations of dipeptide branches connected *via* a branching diamino acid, can be obtained in good yields by solid-phase peptide synthesis and has repeatedly turned out to be optimal in various studies on enzyme models,^33–36^ drug delivery^37^ and antibacterial dendrimers.^38,39^

Introducing a lipid at the dendrimer core led to DMH13 acting as single component transfection reagent for siRNA.^17,18^ This lipidation approach was used to modify G123KL to obtain Z22 as single component transfection dendrimer for pDNA also showing transfection activity on tissue models.^16^ Similar architectures combining a lipidated core with polycationic branches have been reported with poly(amidoamine) (PAMAM) dendrimers for siRNA transfection^40–42^ and antibacterial activities.^43–45^

The optimized siRNA transfection dendrimer DMH13 featured lys-leu branches at G2 and G3, a hydrophobic leu–leu dipeptide in G1, and two side chain palmitoylated lysines at the core, leading to a rather hydrophobic and strongly self-aggregating dendrimer, whereby self-aggregation proved essential for siRNA transfection by DMH13, with a slight advantage of the d-enantiomer over its l-enantiomer in terms of efficiency. Furthermore, the arginine containing analog of DMH13 had been found to strongly bind and internalize siRNA into cells but was unable to release its cargo. By contrast, the present SAR study around dendrimer Z22 showed that pDNA transfection was strongly increased when using arginines instead of lysines in the branches, a full d-enantiomeric sequence with the arg–leu dipeptide repeated across all branches, and only a single side chain stearoylated lysine at the dendrimer core without cysteine, leading to Z34 as the best performing dendrimer for pDNA transfection.

The strong requirement for d-enantiomeric residues in our pDNA transfection dendrimer Z34 was explained by proteolytic degradation, an effect which was much less pronounced in our previously reported siRNA transfection dendrimer DMH13. This might indicate that the pDNA complexes with Z34 are not as tight as the siRNA complexes with DMH13 and therefore more sensitive to protease degradation. The same effect might explain the advantage of arginines over lysines for pDNA transfection. Indeed, arginine residues in the dendrimer branches most likely form bidentate hydrogen bonds with the phosphodiester backbone stabilizing the relatively loose pDNA-Z34 complexes. In siRNA transfection by contrast, arginine analogs of DMH13 were inactive because they bound their cargo too tightly. Dendrimer complexation might also help in protecting pDNA from degradation by nucleases during the transfection process,^46^ a protection which is probably more critical than for siRNA which is more shielded than pDNA.

The observed inhibition of dendrimer mediated pDNA transfection by bafilomycin A1 and the restoration of the activity of l-enantiomeric transfection dendrimers in the presence of protease inhibitors provide strong evidence that pDNA–dendrimer complexes enter cells by endocytosis, and that the full dendrimer structure is required for transfection. Furthermore, the absence of any effect of dexamethasone on transfection efficiency suggests that the nuclear membrane does not represent a barrier, which might indicate that the dendrimers also help nuclear entry by permeabilizing the nuclear envelope. In any event, the expression of GFP encoded by the plasmid as a sign of successful transfection as well as the confirmation of gene editing show that Z34 is a functional transfection reagent for CRISPR/Cas9 pDNA.

## Conclusion

In summary, the SAR study reported here highlighted the structural features necessary for CRISPR/Cas9 pDNA transfection by our peptide dendrimer Z22, and resulted in a simpler and more active analog, dendrimer Z34, shown to induce functional gene editing. Compared to polymeric and lipid-based transfection reagents, the key advantage of peptide dendrimers such as Z34 lies in their entirely well-defined chemical structure which is accessible reproducibly in pure form by standard solid-phase peptide synthesis and HPLC purification. The possibility to vary the dendrimer sequence offers possibilities to fine-tune it for various applications. The versatility of our dendrimer platform should facilitate its further development for non-viral transfection of nucleic acids.

## Author contributions

SZ designed and carried out the study. EZ performed transfection experiments for CRISPR/Cas9 gene editing and dendrimer aggregation experiments. LB designed, performed and analyzed gene editing experiments. FH co-designed and analyzed gene editing experiments. TD and JLR co-designed and co-supervised the study and wrote the paper.

## Data availability

The data supporting this article have been included as part of the ESI.†

## Conflicts of interest

There are no conflicts to declare.

## Supplementary Material

CB-005-D4CB00116H-s001
