# Supplementary material for: Peptide dendrimers transfecting CRISPR/Cas9 plasmid DNA: optimization and mechanism
Source: RSC Chem Biol. 2024 Jul 29;5(9):891–900. doi: 10.1039/d4cb00116h (PMC11352961; doi:10.1039/d4cb00116h)
Supplement: CB-005-D4CB00116H-s001 [file CB-005-D4CB00116H-s001.pdf]

Supplementary Information (SI) for RSC Chemical Biology.
This journal is © The Royal Society of Chemistry 2024

Supporting information for:

Peptide dendrimers transfecting CRISPR/Cas9 plasmid DNA: optimization and mechanism

Susanna Zamolo,[a)] Elena Zakharova,[a)] Lise Boursinhac,[b)] Florian Hollfelder,[b)] Tamis Darbre[a)]* and Jean-Louis Reymond[a)]*

[a)] *Department of Chemistry, Biochemistry and Pharmaceutical Sciences, University of Bern, Freiestrasse 3, 3012 Bern, Switzerland;*

[b)] *Department of Biochemistry, University of Cambridge, 80 Tennis Court Road, Cambridge CB2 1GA, United Kingdom*

*E-mail: tamis.darbre@unibe.ch

*E-mail: jean-louis.reymond@unibe.ch

Table of content

1. Dendrimer synthesis and characterization 2
2. Biology 19
  2.1. pDNA transfection and transgene expression assay (GFP) 19
  2.2. Cell viability by PrestoBlue™ assay 20
  2.3. pDNA transfection in presence of PDI activator or inhibitor 20
  2.4. Critical micellar concentration (CMC) 21
  2.5. Free pDNA assay by Quant-iT™ Picogreen® 21
  2.6. Internalization assay 22
  2.7. Confocal microscopy 22
  2.8. pDNA transfection in presence of Bafilomycin A1 23
  2.9. Stability to proteinase K 23
  2.10. pDNA transfection in presence of protease inhibitors 25
  2.11. pDNA transfection in presence of Dexamethasone 25
  2.12. Measure of SpCas9 cleavage activity after pDNA* transfection by TIDE assay 25
3. References 29

1. Dendrimer synthesis and characterization

All reagents, salts and buffers used were purchased from Sigma Aldrich, Fluorochem Ltd, Iris Biotech Gmbh, TCI (Tokyo Chemical Company), GL Biochem, with synthesis grade purity as specified by each supplier. Amino acids were used as the following derivatives: Fmoc-Ala-OH, Fmoc-Cys(Trt)-OH, Fmoc-Leu-OH, Fmoc-D-Leu-OH, Fmoc-Lys(Boc)-OH, Fmoc-D-Lys(Boc)-OH, Fmoc-Lys(Fmoc)-OH, Fmoc-D-Lys(Fmoc)-OH, Fmoc-Lys(Alloc)-OH, Fmoc-D-Lys(Alloc)-OH, Fmoc-Lys(Mtt)-OH, Fmoc-D-Lys(Mtt)-OH, Fmoc-Arg(Pbf)-OH and were purchased from Iris Biotech GmbH or GL Biochem. Rink Amide AM resin LL was purchased from Novabiochem. Peptide dendrimers synthesis was performed manually in polypropylene syringes fitted with a polyethylene frit, a Teflon stopcock and stopper or automatically by CEM Liberty Blue Automated Microwave Peptide Synthesizer.

Analytical RP-HPLC was performed with an Ultimate 3000 Rapid Separation LC-MS System (DAD-3000RS diode array detector) using an Acclaim RSLC 120 C18 column (2.2 μm, 120 Å, 3×50 mm, flow 1.2 mL/min) from Dionex. Data recording and processing was done with Dionex Chromeleon Management System Version 6.80 (analytical RP-HPLC). All RP-HPLC were using HPLC-grade acetonitrile and Milli-Q deionized water. The elution solutions were: A Milli-Q deionized water containing 0.05% TFA; D Milli-Q deionized water/acetonitrile (10:90, v/v) containing 0.05% TFA. Preparative RP-HPLC was performed with a Waters automatic Prep LC Controller System containing the four following modules: Waters2489 UV/Vis detector, Waters2545 pump, Waters Fraction Collector III and Waters 2707 Autosampler. A Dr. Maisch GmbH Reprospher column (C18-DE, 100×30 mm, particle size 5 μm, pore size 100 Å, flow rate 40 mL/min) was used. Compounds were detected by UV absorption at 214 nm using a Waters 248 Tunable Absorbance Detector. Data recording and processing was performed with Waters ChromScope version 1.40 from Waters Corporation. All RP-HPLC were using HPLC-grade acetonitrile and Milli-Q deionized water. The elution solutions were: A: Milli-Q deionized water containing 0.1% TFA; D: Milli-Q deionized water/acetonitrile (10:90, v/v) containing 0.1% TFA. MS spectra were recorded on a Thermo Scientific LTQ OrbitrapXL. MS spectra were provided by the MS analytical service of the Department of Chemistry and Biochemistry at the University of Bern (group PD Dr. Stefan Schürch).

Peptide dendrimers were synthesized according to the previously reported procedure.[1]

Table S1: Yield and ESI-MS data for the synthesized peptide dendrimers after preparative HPLC purification

no.	Sequence[a)]	Yield[b)] mg (%)	MS[c)] calc/obs
**Z23**	(RL)$_8$(*K*RL)$_4$(*K*RL)$_2$*K*K(C$_{18}$)	30 (2.8)	5076.64/5076.64
**Z24**	(RL)$_8$(*K*RL)$_4$(*K*RL)$_2$*K*K(C$_{18}$)C	25 (2.3)	5179.65/5179.65
**Z25**	(RL)$_8$(*K*RL)$_4$(*K*RL)$_2$*K*K(C$_{18}$)A	51 (4.7)	5147.68/4147.68
**Z26**	(kl)$_8$(*k*kl)$_4$(*k*kl)$_2$*k*k(C$_{18}$)	19 (7.6)	4684.55/4684.56
**Z27**	(kl)$_8$(*k*kl)$_4$(*k*kl)$_4$*k*k(C$_{18}$)k(C$_{18}$)	35 (2.6)	5078.91/5078.91
**Z28**	(kl)$_8$(*k*kl)$_4$(*k*kl)$_2$*k*lllll	122 (9.5)	4855.62/4855.63
**Z29**	(kl)$_8$(*k*kl)$_4$(*k*kl)$_2$*k*k(C$_{16}$)	22 (8.6)	4656.52/4656.52
**Z30**	(kl)$_8$(*k*kl)$_4$(*k*kl)$_2$*k*k(C$_{18}$)a	114 (9)	4755.59/4755.59
**Z31**	(kl)$_8$(*k*kl)$_4$(*k*ll)$_2$*k*k(C$_{18}$)	64 (5.3)	4654.53/4654.53
**Z32**	(kl)$_8$(*k*ll)$_4$(*k*ll)$_2$*k*k(C$_{18}$)	65 (5.8)	4594.49/4594.48
**Z33**	(rl)$_8$(*k*rl)$_4$(*k*rl)$_2$*k*k(C$_{16}$)	13.5 (5.1)	5048.61/5048.60
**Z34**	(rl)$_8$(*k*rl)$_4$(*k*rl)$_2$*k*k(C$_{18}$)	12 (4.7)	5076.64/5076.63
**Z35**	(rl)$_8$(*k*rl)$_4$(*k*rl)$_2$*k*k(C$_{18}$)a	25 (2.3)	5147.68/5147.68
**Z36**	(rl)$_8$(*k*rl)$_4$(*k*ll)$_2$*k*k(C$_{18}$)	44 (4.3)	4990.61/4990.61
**Z37**	(rl)$_8$(*k*ll)$_4$(*k*ll)$_2$*k*k(C$_{18}$)	21 (2.3)	4818.54/4818.54

a) One-letter code amino acids are used, *K*(*k*) is the branching lysine residue, C-termini are carboxamide $CONH_2$, and all N-termini are free. Alkyl chains in the structure are represented by "C" followed by their number of carbon atoms. b) Isolated yields as trifluoroacetate salt after preparative HPLC purification. c) ESI-MS positive mode.

Z23 (RL)$_8$(*K*RL)$_4$(*K*RL)$_2$*K*K(C$_{18}$) was obtained after manual synthesis as foamy colorless solid after preparative RP-HPLC (30 mg, 3.9 µmol, 3%). Analytical RP-HPLC: t_R= 3.86 min (100% A to 100% D in 7.5 min, λ= 214 nm). MS (ESI+):$C_{234}H_{455}N_{87}O_{37}$ calc./obs. 5076.64/5076.64

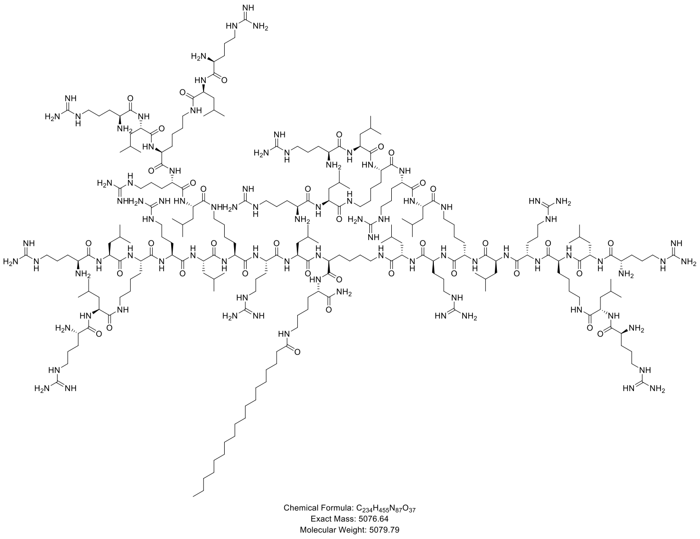

LCMS

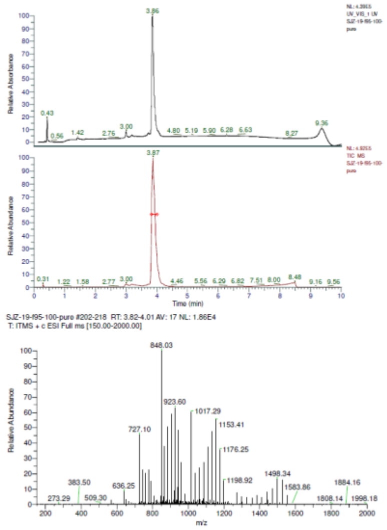

MASS SPECTRUM, HRMS (NSI+):

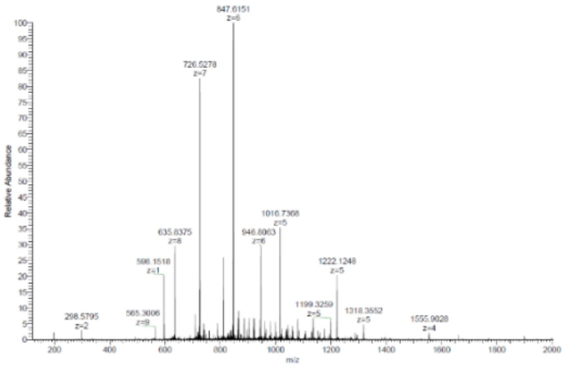

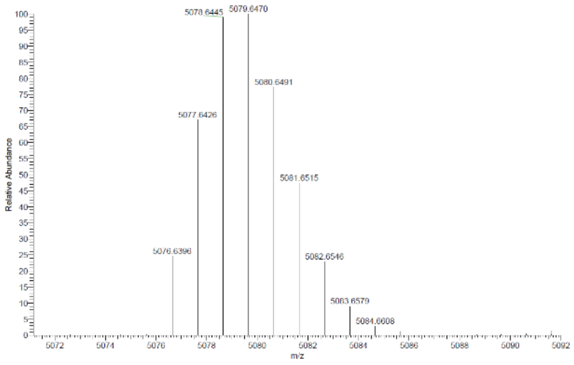

Z24 (RL)$_8$(*K*RL)$_4$(*K*RL)$_2$*K*K(C$_{18}$)C was obtained after manual synthesis as foamy colorless solid after preparative RP-HPLC (25 mg, 3.3 µmol, 3%). Analytical RP-HPLC: t_R= 3.89 min (100% A to 100% D in 7.5 min, λ= 214 nm). MS (ESI+):$C_{237}H_{460}N_{88}O_{38}S$ calc./obs. 5179.65/5179.65

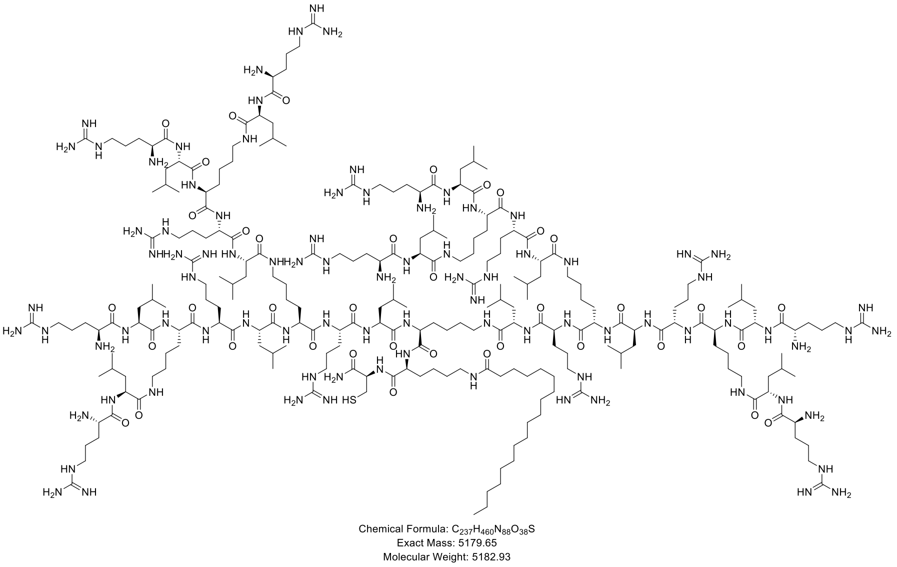

LCMS

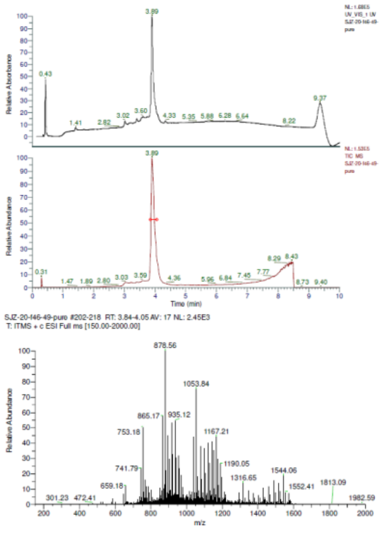

MASS SPECTRUM, HRMS (NSI+):

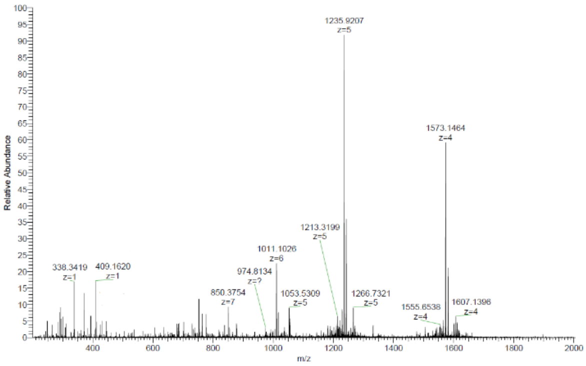

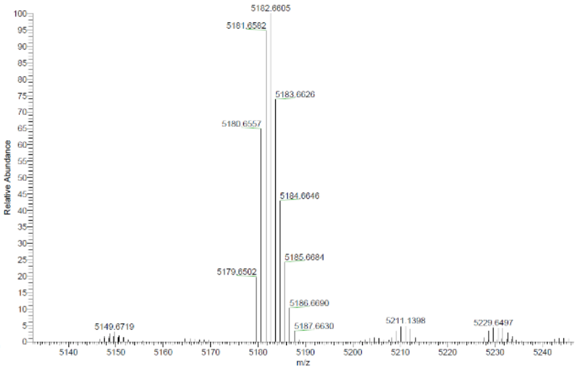

Z25 (RL)$_8$(*K*RL)$_4$(*K*RL)$_2$*K*K(C$_{18}$)A was obtained after manual synthesis as foamy colorless solid after preparative RP-HPLC (51 mg, 6.7 µmol, 5%). Analytical RP-HPLC: t_R= 3.85 min (100% A to 100% D in 7.5 min, λ= 214 nm). MS (ESI+):$C_{237}H_{460}N_{88}O_{38}$ calc./obs. 5147.68/4147.68

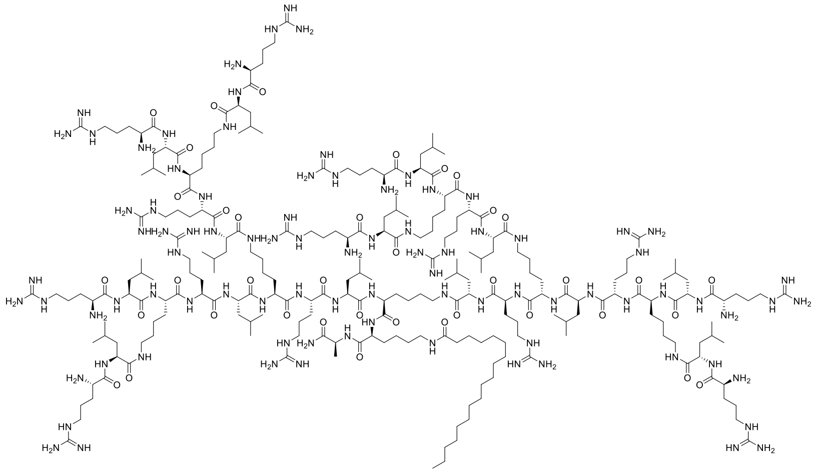

Chemical Formula: $C_{237}H_{460}N_{88}O_{38}$
Exact Mass: 5147.68
Molecular Weight: 5150.86

LCMS

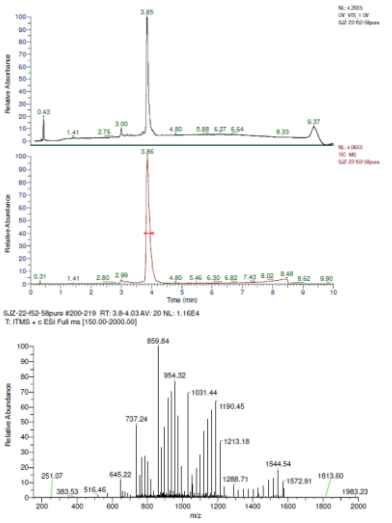

MASS SPECTRUM, HRMS (NSI+):

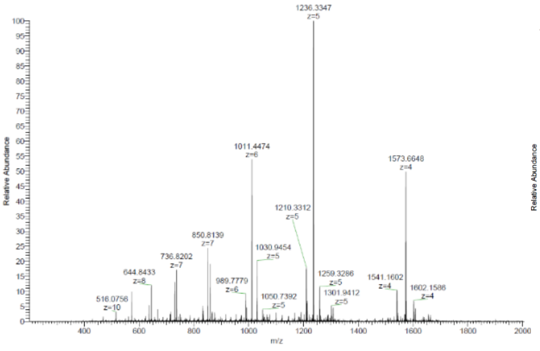

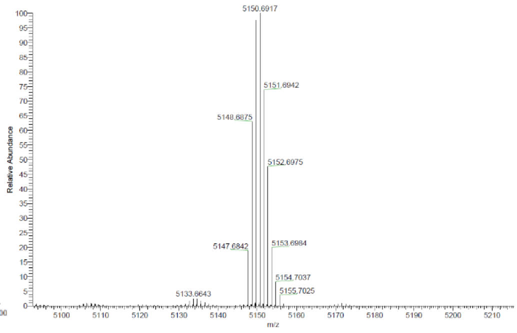

Z26 (kl)$_8$(*k*kl)$_4$(*k*kl)$_2$*k*k(C$_{18}$) was obtained after manual synthesis as foamy colorless solid after preparative RP-HPLC (19 mg, 2.6 µmol, 8%). Analytical RP-HPLC: t_R= 3.76 min (100% A to 100% D in 7.5 min, λ= 214 nm). MS (ESI+):$C_{234}H_{455}N_{59}O_{37}$ calc./obs. 4684.55/4684.56

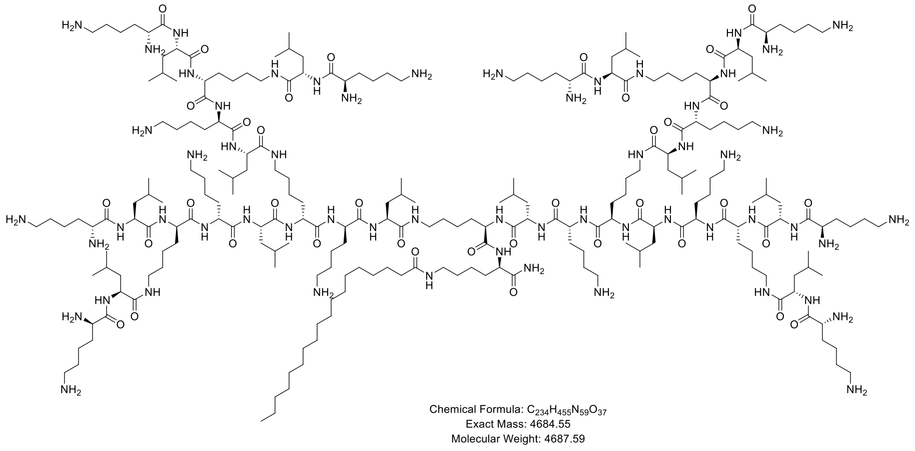

LCMS

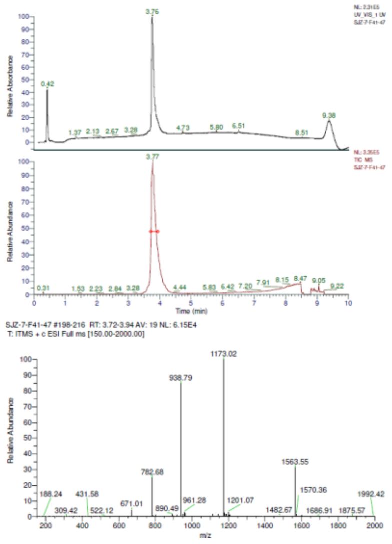

MASS SPECTRUM, HRMS (NSI+):

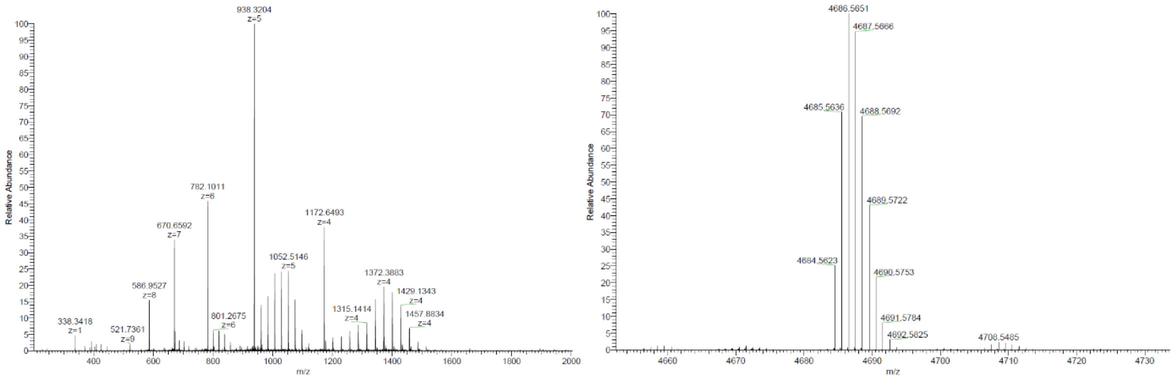

Z27 $(kl)_8(\boldsymbol{k}kl)_4(\boldsymbol{k}kl)_4\boldsymbol{k}k(C_{18})k(C_{18})$ was obtained after manual synthesis as foamy colorless solid after preparative RP-HPLC (35 mg, 6.9 µmol, 3%). Analytical RP-HPLC: t_R= 4.99 min (100% A to 100% D in 7.5 min, λ= 214 nm). MS (ESI+):$C_{258}H_{501}N_{61}O_{39}$ calc./obs. 5078.91/5078.91

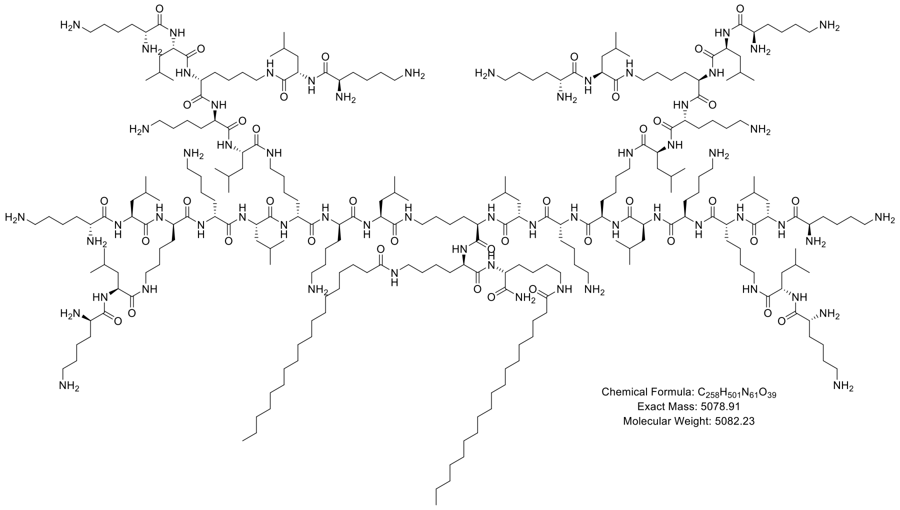

LCMS

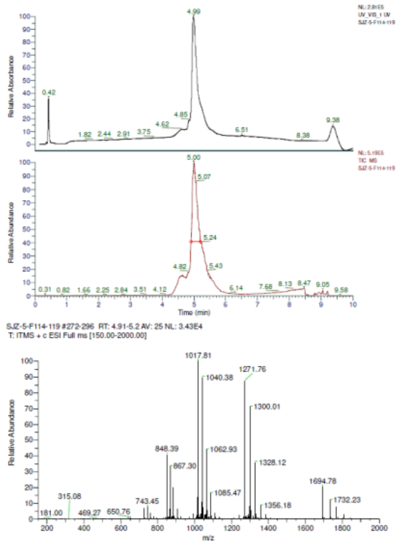

MASS SPECTRUM, HRMS (NSI+):

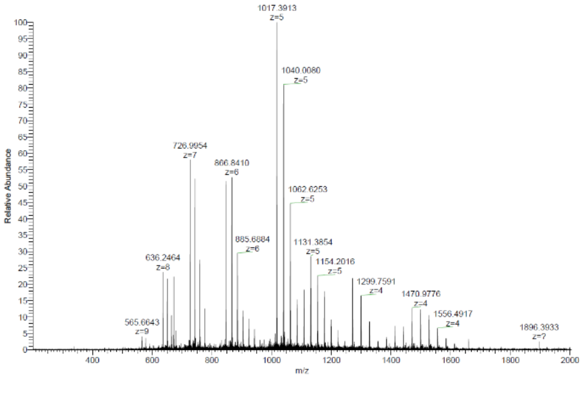

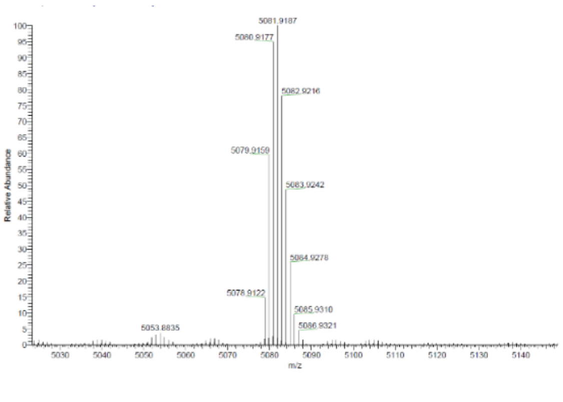

Z28 (kl)$_8$(*k*kl)$_4$(*k*kl)$_2$*k*lllll was obtained after manual synthesis as foamy colorless solid after preparative RP-HPLC (122 mg, 16.6 µmol, 10%). Analytical RP-HPLC: t_R= 3.22 min (100% A to 100% D in 7.5 min, λ= 214 nm). MS (ESI+):$C_{240}H_{464}N_{62}O_{40}$ calc./obs. 4855.62/4855.63

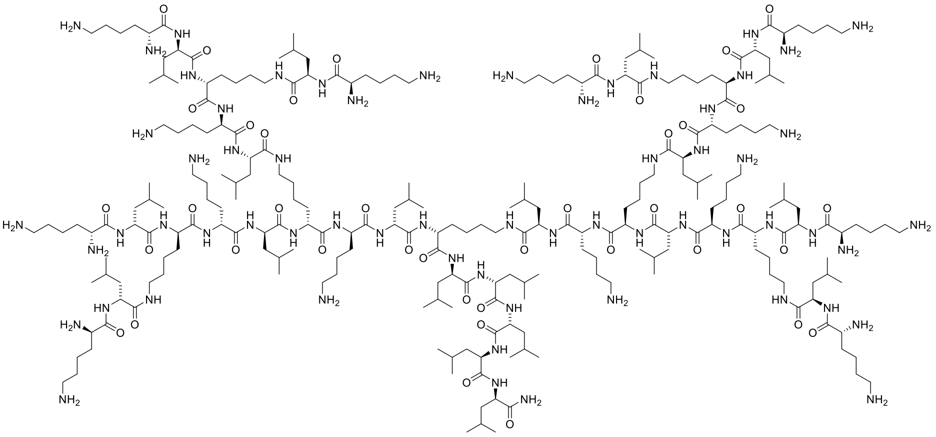

Chemical Formula: $C_{240}H_{464}N_{62}O_{40}$
Exact Mass: 4855.62
Molecular Weight: 4858.75

LCMS

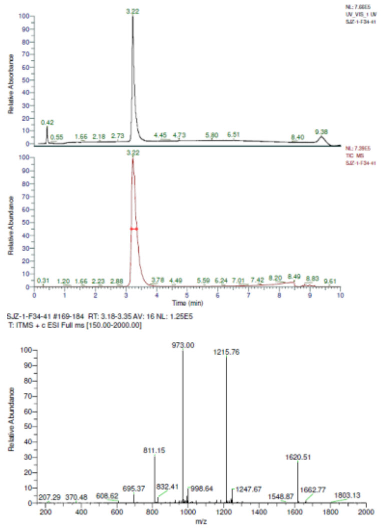

MASS SPECTRUM, HRMS (NSI+):

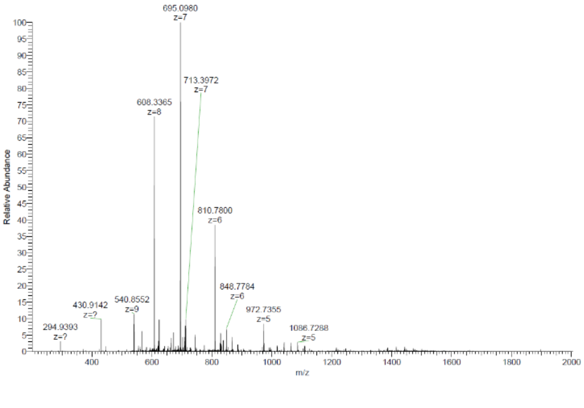

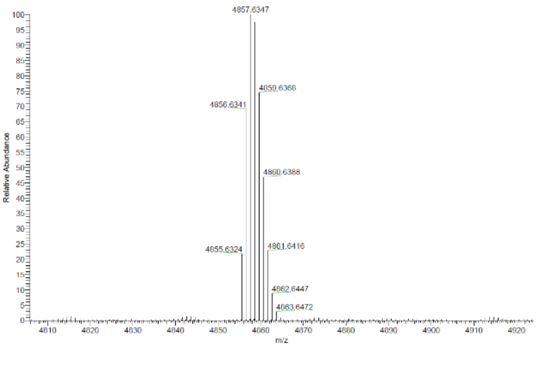

Z29 (kl)$_8$(*k*kl)$_4$(*k*kl)$_2$*k*k(C$_{16}$) was obtained after manual synthesis as foamy colorless solid after preparative RP-HPLC (22 mg, 3.1 µmol, 9%). Analytical RP-HPLC: t_R= 3.60 min (100% A to 100% D in 7.5 min, λ= 214 nm). MS (ESI+):$C_{232}H_{451}N_{59}O_{37}$ calc./obs. 4656.52/4656.52

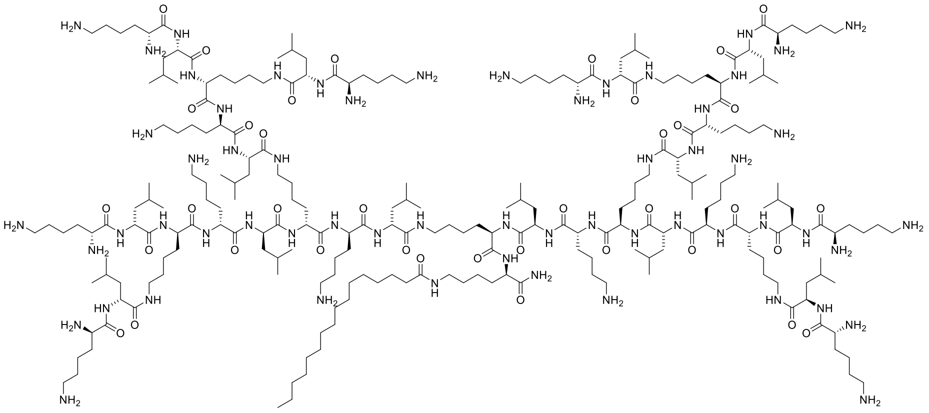

Chemical Formula: $C_{232}H_{451}N_{59}O_{37}$
Exact Mass: 4656.52
Molecular Weight: 4659.54

LCMS

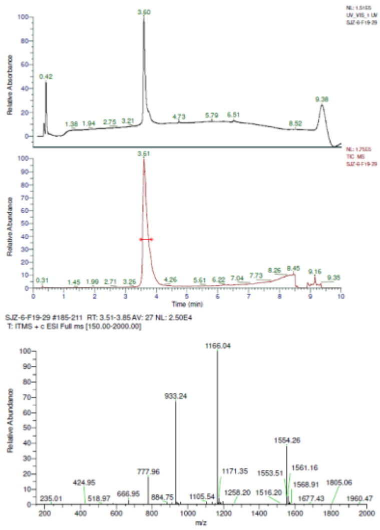

MASS SPECTRUM, HRMS (NSI+):

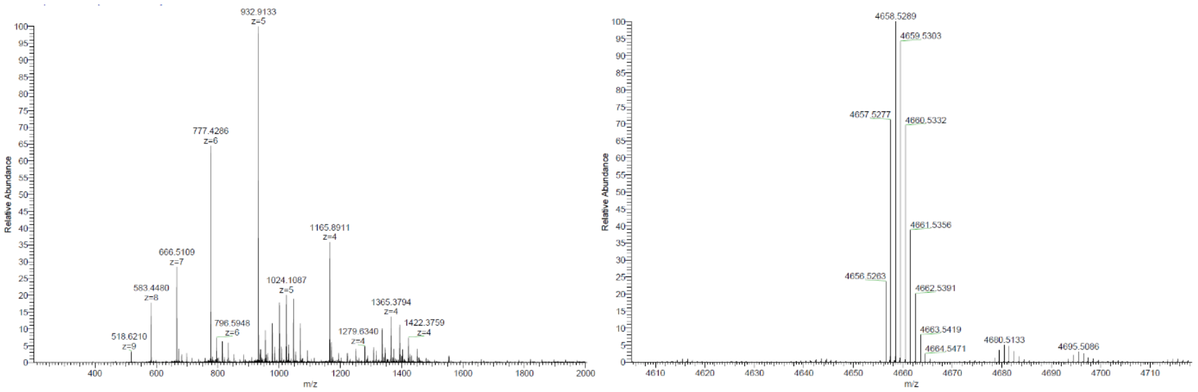

Z30 (kl)$_8$(*k*kl)$_4$(*k*kl)$_2$*k*k(C$_{18}$)a was obtained after manual synthesis as foamy colorless solid after preparative RP-HPLC (114 mg, 15.7 µmol, 9%). Analytical RP-HPLC: t_R= 3.66 min (100% A to 100% D in 7.5 min, λ= 214 nm). MS (ESI+):$C_{237}H_{460}N_{60}O_{38}$ calc./obs. 4755.59/4755.59

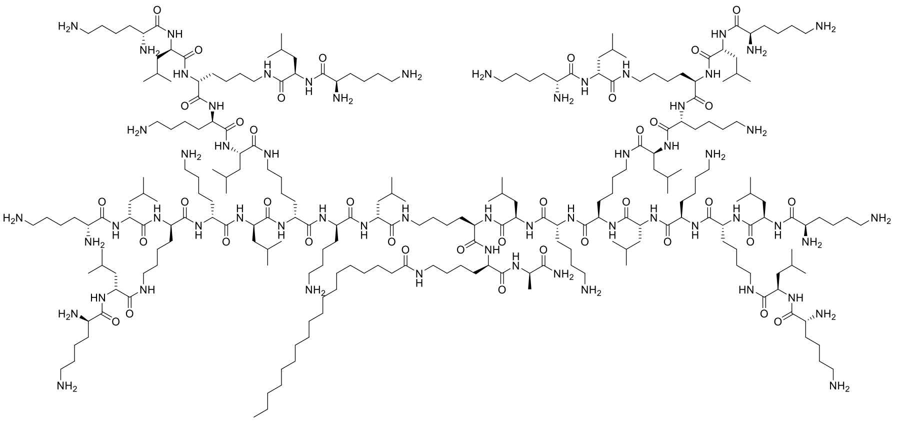

Chemical Formula: $C_{237}H_{460}N_{60}O_{38}$
Exact Mass: 4755.59
Molecular Weight: 4758.67

LCMS

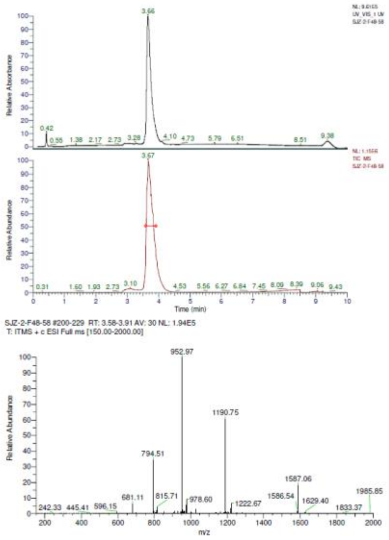

MASS SPECTRUM, HRMS (NSI+):

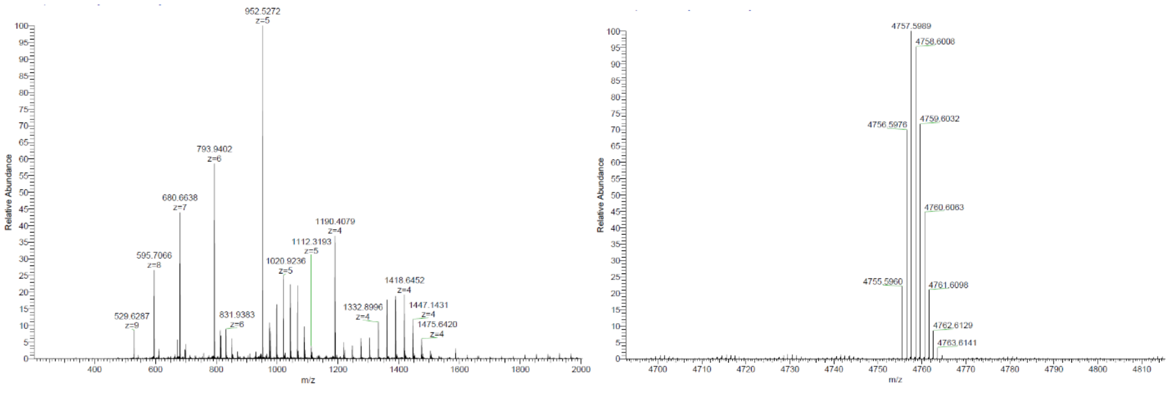

Z31 (kl)$_8$(*k*kl)$_4$(*k*ll)$_2$*k*k(C$_{18}$) was obtained after manual synthesis as foamy colorless solid after preparative RP-HPLC (64 mg, 9.2 µmol, 5%). Analytical RP-HPLC: t_R= 3.94 min (100% A to 100% D in 7.5 min, λ= 214 nm). MS (ESI+):$C_{234}H_{453}N_{57}O_{37}$ calc./obs. 4654.53/4654.53

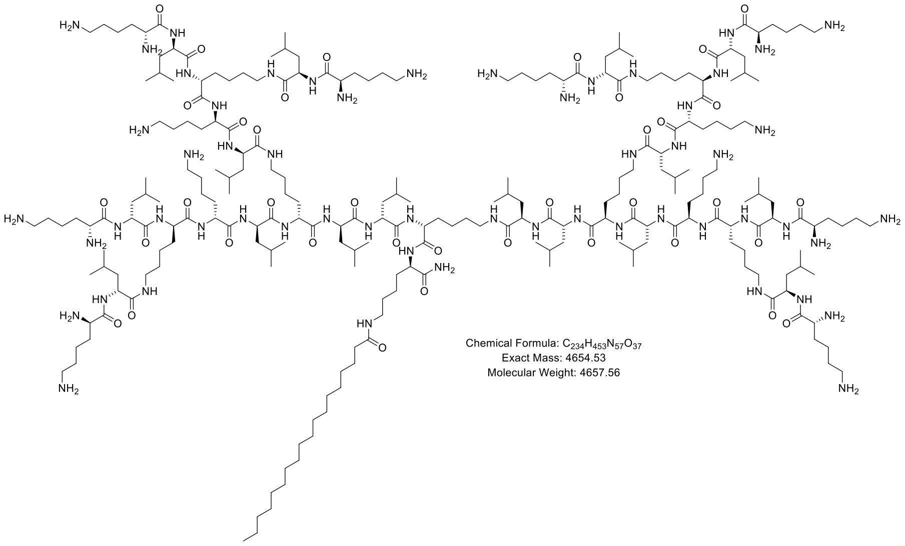

LCMS

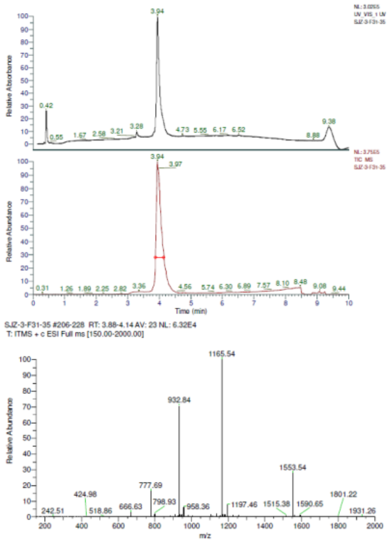

MASS SPECTRUM, HRMS (NSI+):

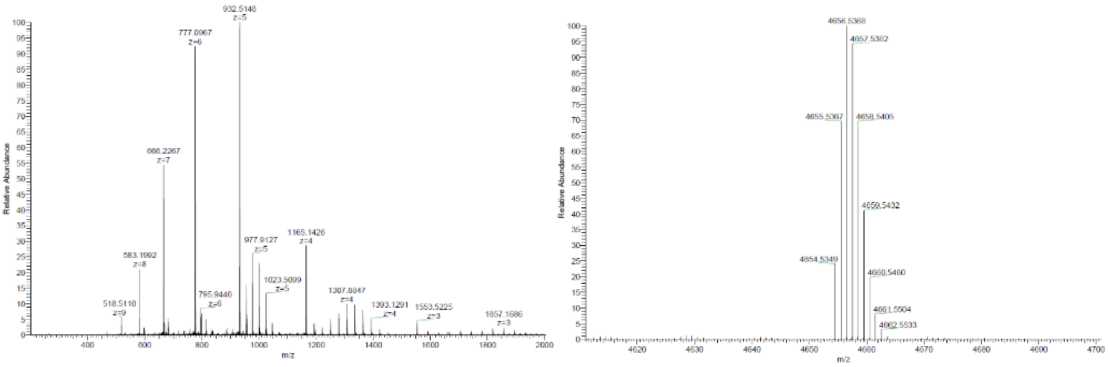

Z32 (kl)$_8$(*k*ll)$_4$(*k*ll)$_2$*k*k(C$_{18}$) was obtained after manual synthesis as foamy colorless solid after preparative RP-HPLC (65 mg, 10.1 µmol, 6%). Analytical RP-HPLC: t_R= 4.56 min (100% A to 100% D in 7.5 min, λ= 214 nm). MS (ESI+):$C_{234}H_{449}N_{53}O_{37}$ calc./obs. 4594.49/4594.48

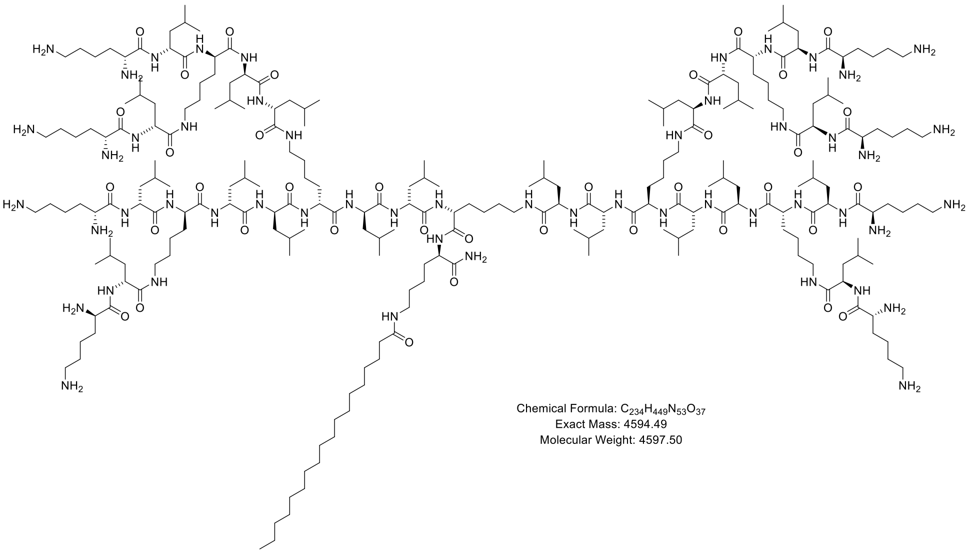

LCMS

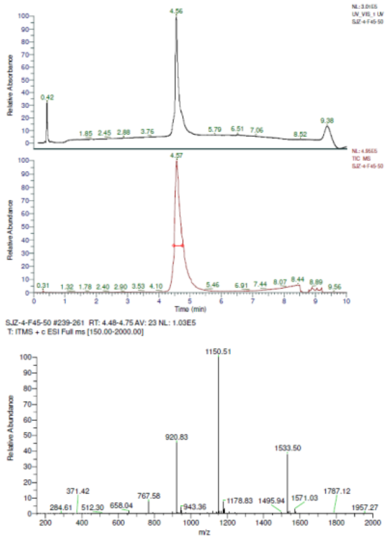

MASS SPECTRUM, HRMS (NSI+):

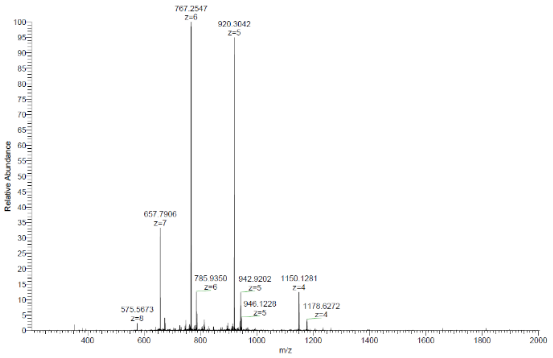

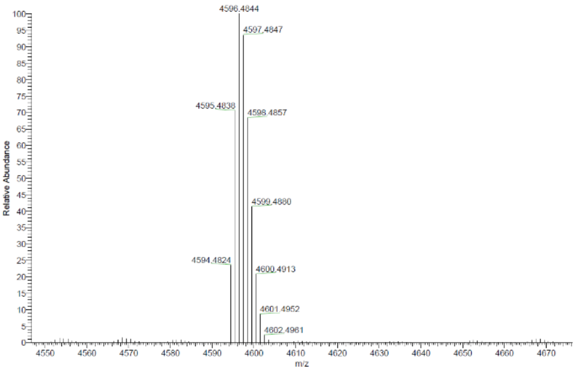

Z33 (rl)$_8$(*k*rl)$_4$(*k*rl)$_2$*k*k(C$_{16}$) was obtained after manual synthesis as foamy colorless solid after preparative RP-HPLC (13.5 mg, 1.8 µmol, 5%). Analytical RP-HPLC: t_R= 3.69 min (100% A to 100% D in 7.5 min, λ= 214 nm). MS (ESI+):$C_{232}H_{451}N_{87}O_{37}$ calc./obs. 5048.61/5048.60

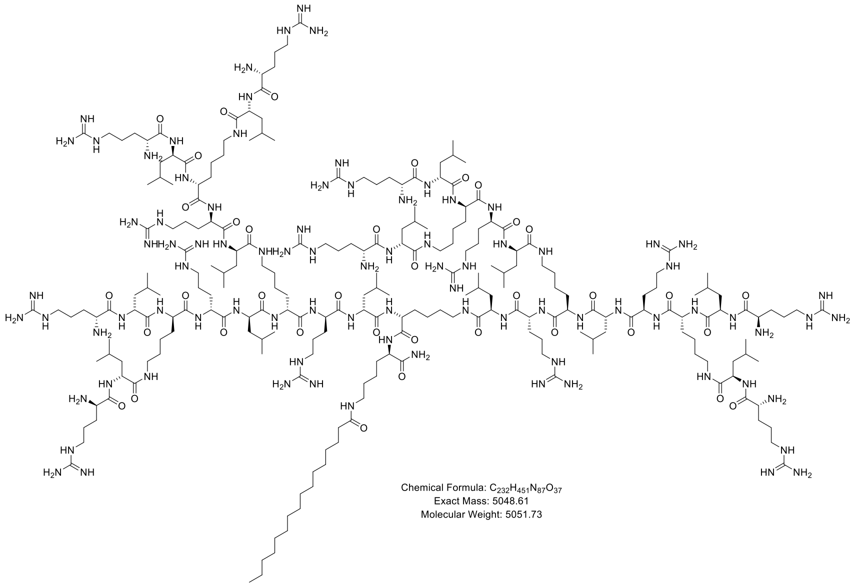

LCMS

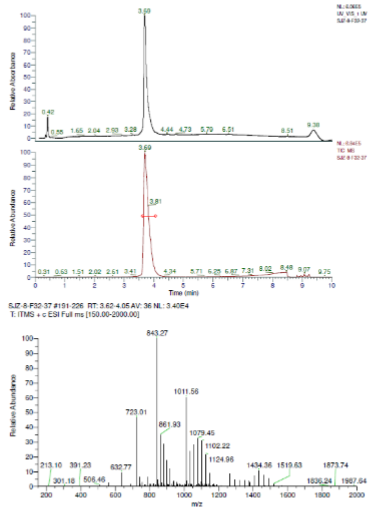

MASS SPECTRUM, HRMS (NSI+):

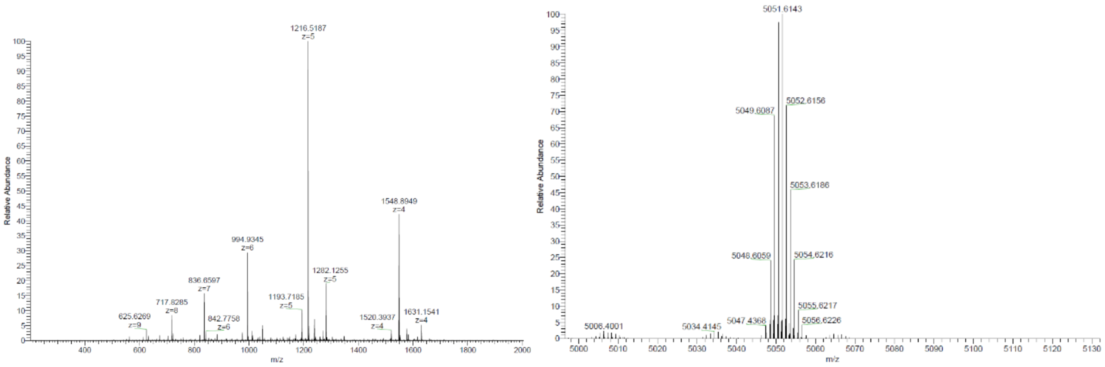

Z34 (rl)$_8$(*k*rl)$_4$(*k*rl)$_2$*k*k(C$_{18}$) was obtained after manual synthesis as foamy colorless solid after preparative RP-HPLC (12 mg, 1.6 µmol, 5%). Analytical RP-HPLC: t_R= 3.90 min (100% A to 100% D in 7.5 min, λ= 214 nm). MS (ESI+):$C_{234}H_{455}N_{87}O_{37}$ calc./obs. 5076.64/5076.63

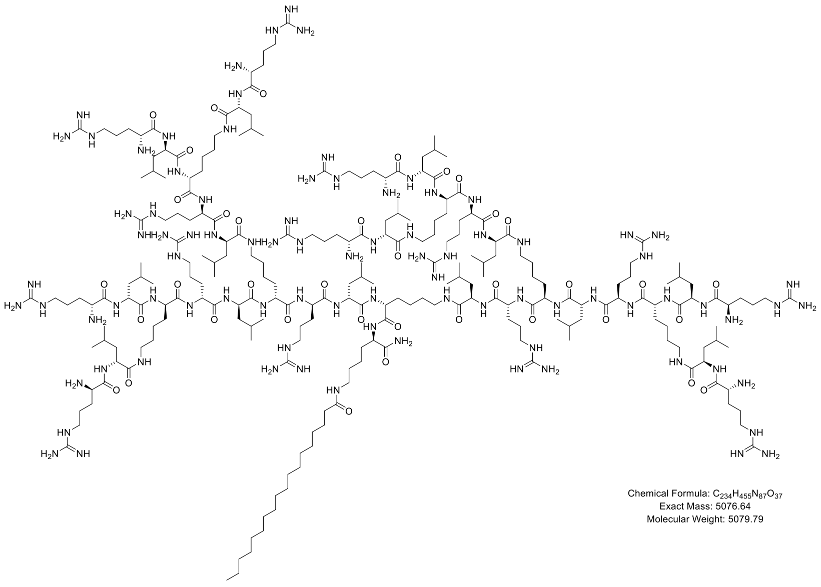

LCMS

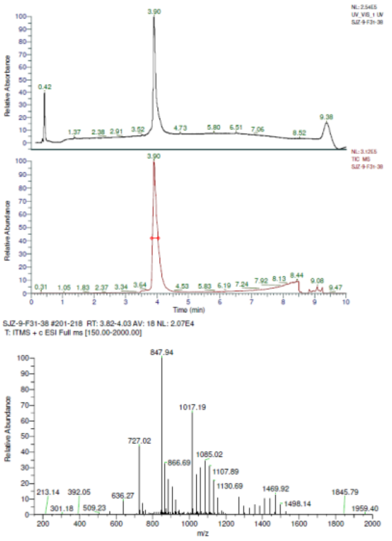

MASS SPECTRUM, HRMS (NSI+):

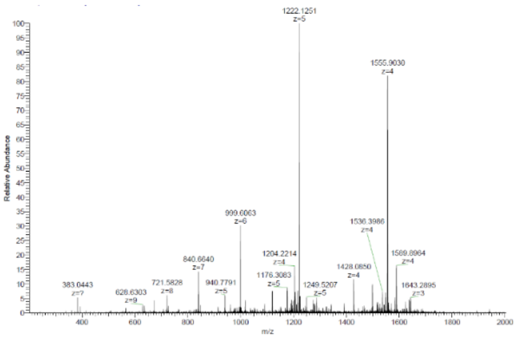

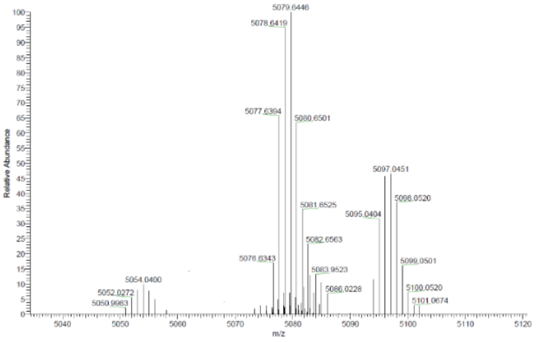

Z35 (rl)₈(*k*rl)₄(*k*rl)₂*k*k(C₁₈)a was obtained after manual synthesis as foamy colorless solid after preparative RP-HPLC (25 mg, 3.3 µmol, 2%). Analytical RP-HPLC: t_R= 3.90 min (100% A to 100% D in 7.5 min, λ= 214 nm). MS (ESI+):$C_{237}H_{460}N_{88}O_{38}$ calc./obs. 5147.68/5147.68

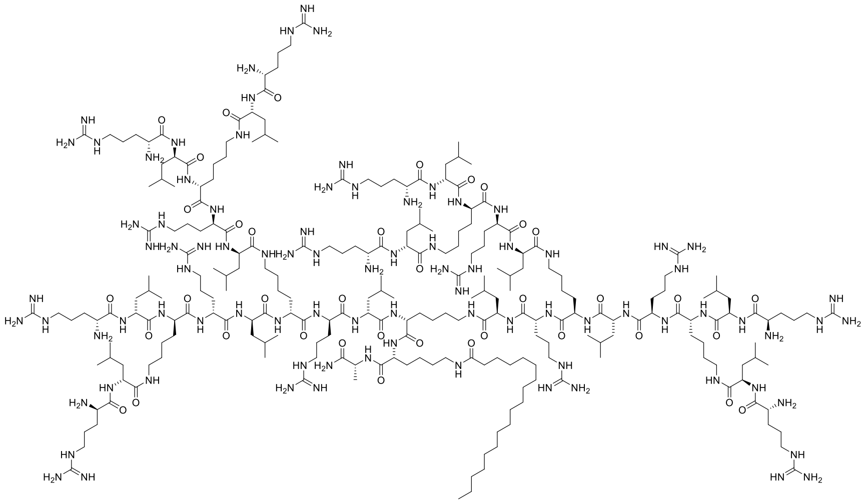

Chemical Formula: $C_{237}H_{460}N_{88}O_{38}$
Exact Mass: 5147.68
Molecular Weight: 5150.86

LCMS

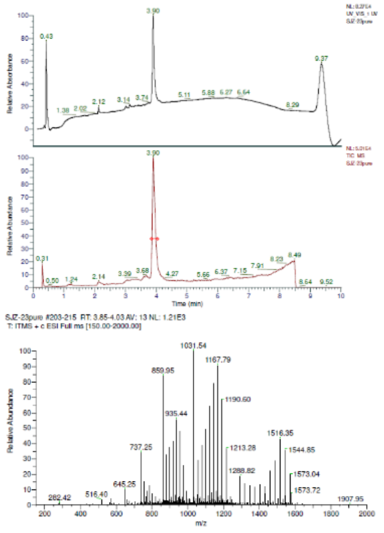

MASS SPECTRUM, HRMS (NSI+):

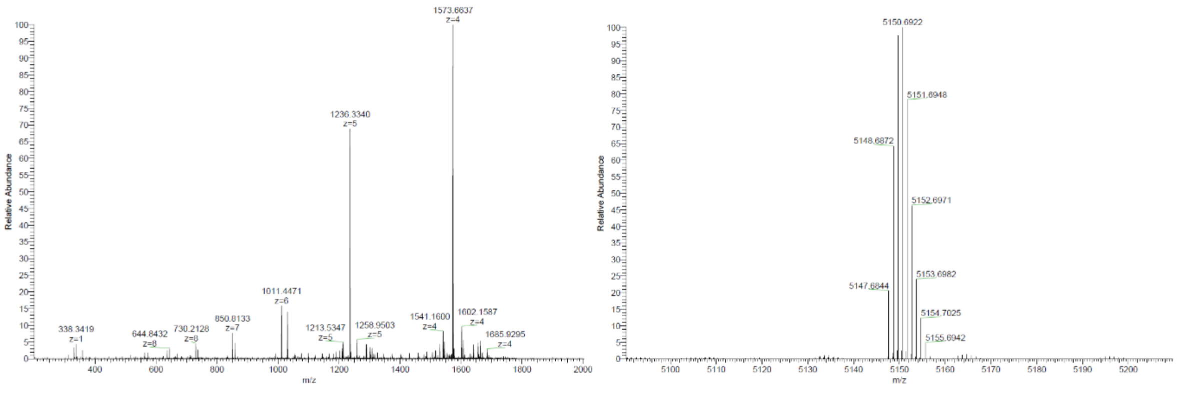

Z36 (rl)$_8$(*k*rl)$_4$(*k*ll)$_2$*k*k(C$_{18}$) was obtained after manual synthesis as foamy colorless solid after preparative RP-HPLC (44 mg, 6.1 µmol, 4%). Analytical RP-HPLC: t_R= 4.05 min (100% A to 100% D in 7.5 min, λ= 214 nm). MS (ESI+):$C_{234}H_{453}N_{81}O_{37}$ calc./obs. 4990.61/4990.61

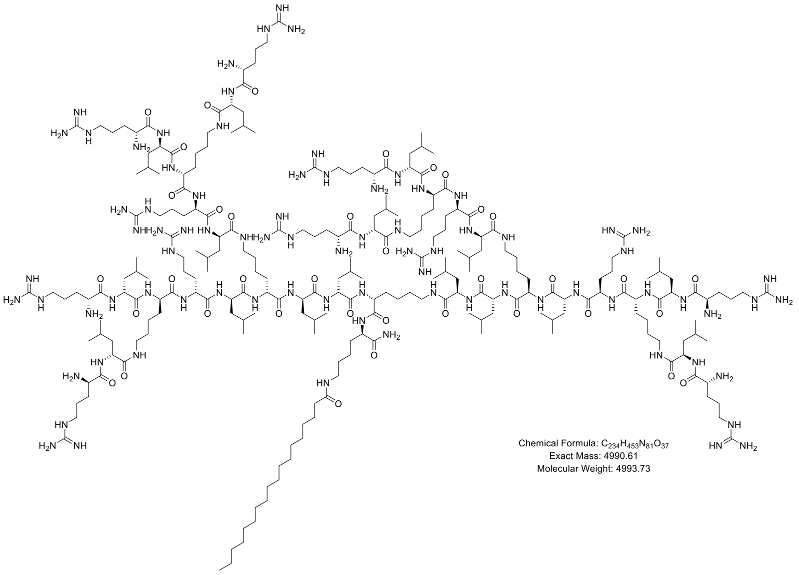

LCMS

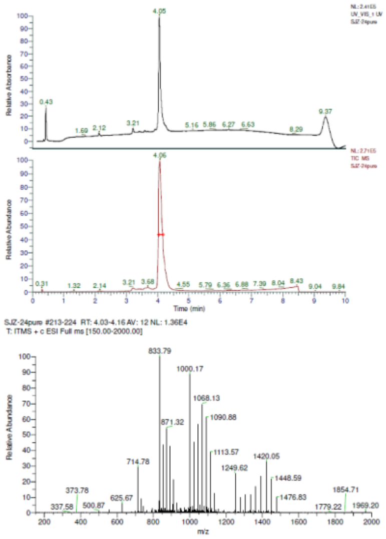

MASS SPECTRUM, HRMS (NSI+):

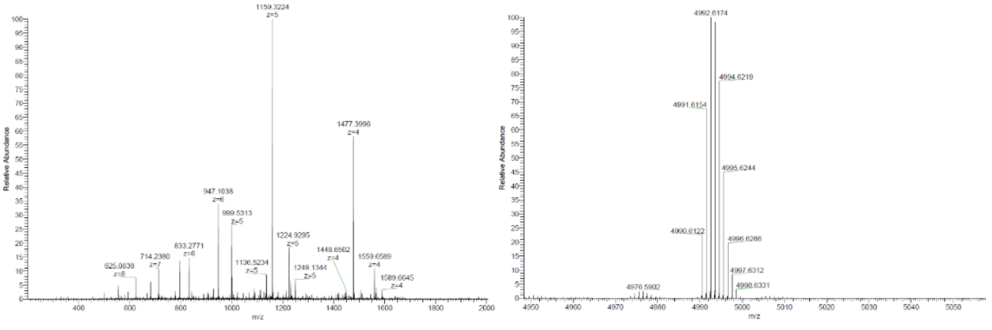

Z37 (rl)$_8$(*k*ll)$_4$(*k*ll)$_2$*k*k(C$_{18}$) was obtained after manual synthesis as foamy colorless solid after preparative RP-HPLC (21 mg, 3.2 µmol, 2%). Analytical RP-HPLC: t_R= 4.67 min (100% A to 100% D in 7.5 min, λ= 214 nm). MS (ESI+):$C_{234}H_{449}N_{69}O_{37}$ calc./obs. 4818.54/4818.54

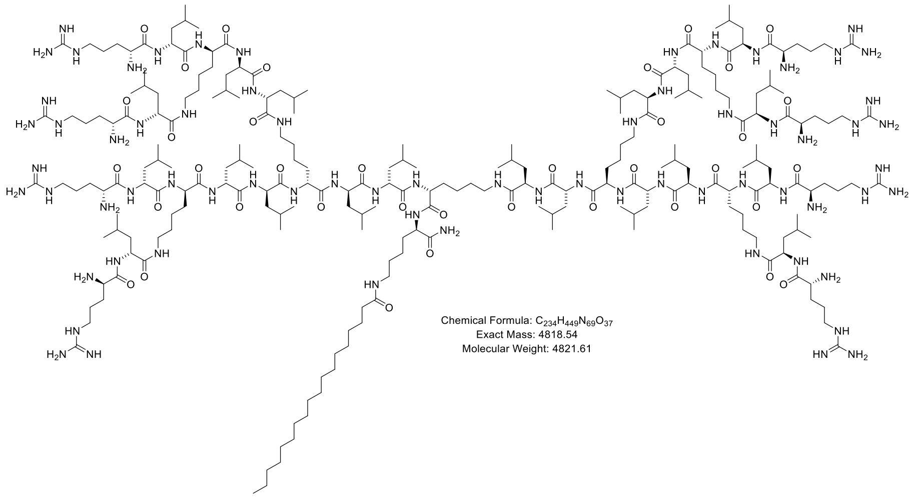

LCMS

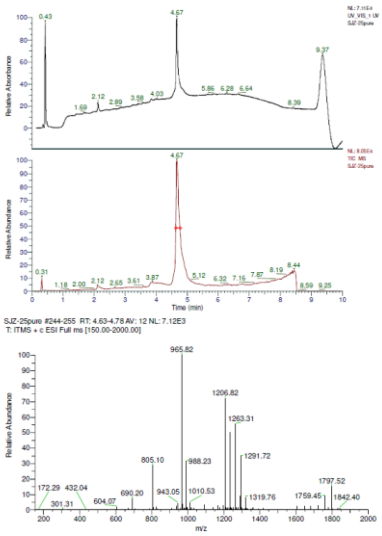

MASS SPECTRUM, HRMS (NSI+):

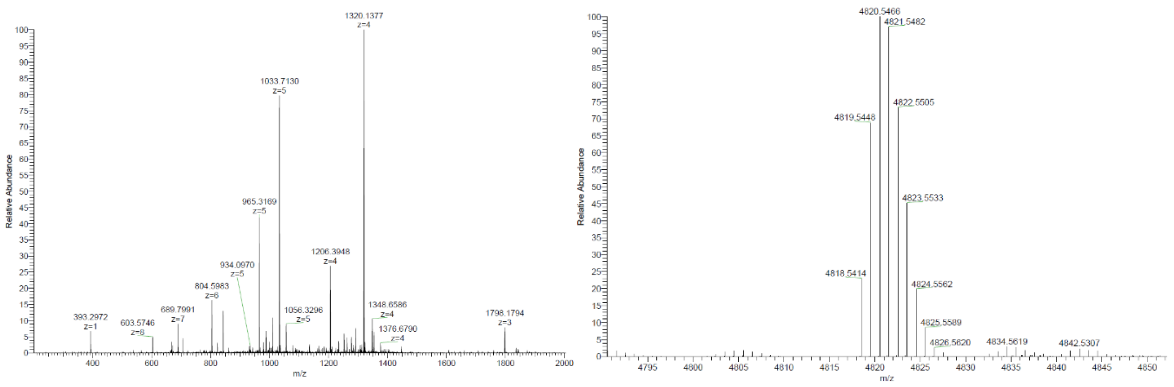

2. Biology

HeLa and HEK-293 cells (ATCC, Manassas, USA) were maintained in DMEM (Thermo Fisher Scientific, Reinach, CH) supplemented with 10% fetal calf serum (FCS, Thermo fisher Scientific) at 37 °C in a humidified atmosphere in 5% carbon dioxide. THP-1 cells (ATCC, Manassas, USA) were maintained in RPMI 1640 Media (Thermo Fisher Scientific, Reinach, CH) supplemented with 10% fetal calf serum (FCS, Thermo fisher Scientific) at 37 °C in a humidified atmosphere in 5% carbon dioxide. The plasmid DNA (pDNA) encoding both for CRISPR/Cas9 and GFP proteins (CRISPR-Cas9-2A-GFP, 9062 bp) was purchased from DNA2.0, ATUM. The plasmid also contains a cassette for expression of a single guide RNA (sgRNA) against GUSB, encoding for the human beta-glucuronidase. Lipofectamine® 2000 (L2000) was obtained from Sigma-Aldrich and used as positive control transfection agent, in accordance with the manufacturer's instructions.

2.1. pDNA transfection and transgene expression assay (GFP)

The day before transfection, HEK and HeLa cells were seeded in TPP 96-well plates (Faust Laborbedarf AG, Schaffhausen), respectively at $45 \cdot 10^3$ and $25 \cdot 10^3$ cells per well, in order to reach 70-90 % confluence. Peptide dendrimers/DNA complexes were formed by mixing the 6.25 µL of dendrimer solution (N/P 5, 1 mg/mL stock solution in Milli-Q water, 175-240 pmol/well, in 6.25 µL of OptiMEM, to achieve 27.9-38.4 µM) with 6.25 µL of plasmid DNA solution (250 ng in 6.25 µL of OptiMEM, 6.8 nM). Transfection control complexes with L2000 were mixed with plasmid DNA (250 ng in 6.25 µL OptiMEM, 6.8 nM) at the respective published recommended concentrations (2:1, v/w, L2000:DNA, 0.5 µL from the 1 mg/mL of commercial solution in 6.25 µL of OptiMEM).[2] These mixtures were incubated in OptiMEM for 30 min at 25°C (12.5 µL, 3.4 nM of pDNA and 14.0-19.2 µM of peptide dendrimer or 40 µg/mL of L2000). The complexes were then gently diluted in OptiMEM or in OptiMEM plus 10% FCS to a final volume of 100 µL per well (final concentration: 0.42 nM of pDNA, 1.75-2.4 µM of peptide dendrimers or 5 µg/mL of L2000). After removing complete media from the cells, the complexes were added to the plates. The plates were incubated for 4 h at 37 °C in a humidified atmosphere in 5% carbon dioxide. Then, the transfection solutions were replaced by full growth media (DMEM high glucose) for 48 h, followed by transfection efficiency analysis.

The cells were washed twice with PBS and incubated with trypsin for 5 min at 37 °C. Then 100 µL of PBS were added to each well and the transfection efficiency was assessed by FACS

analysis, FITC channel (CytoFLEX Flow Cytometer, Beckman Coulter), counting 10000 events per well.

2.2. Cell viability by PrestoBlue^{TM} assay

Cells were transfected in TPP 96-well plates as previously described. Following the transfection, the medium was removed and replaced with 10% PrestoBlue™ (Thermo Fisher Scientific, Reinach, CH) in DMEM supplemented with 10% FCS. Cells were incubated for 30 min at 37° C in a humidified atmosphere in 5% carbon dioxide. Then, plates were measured on a Tecan Infinite M1000 Pro plate reader at λ_{ex}= 560 nm and λ_{em}= 590 nm and values were normalized to the one of untreated cells.

2.3. pDNA transfection in presence of PDI activator or inhibitor

HEK293 cells were pretreated with DTT or DTNB (2mM or 1.2mM respectively) in DMEM supplemented with 10% FCS for 25 min before transfection, in 96-well TPP plates as described above. Cells were then washed with PBS and peptide dendrimers/pDNA complexes were added to cells and incubated for 4 h at 37°C in a humidified atmosphere in 5% carbon dioxide. Then, complexes were removed and replaced with 100 µL of DMEM supplemented with 10% FCS and incubated for 48 h. Transfection efficiency was assessed as described above after a total of 48 h.

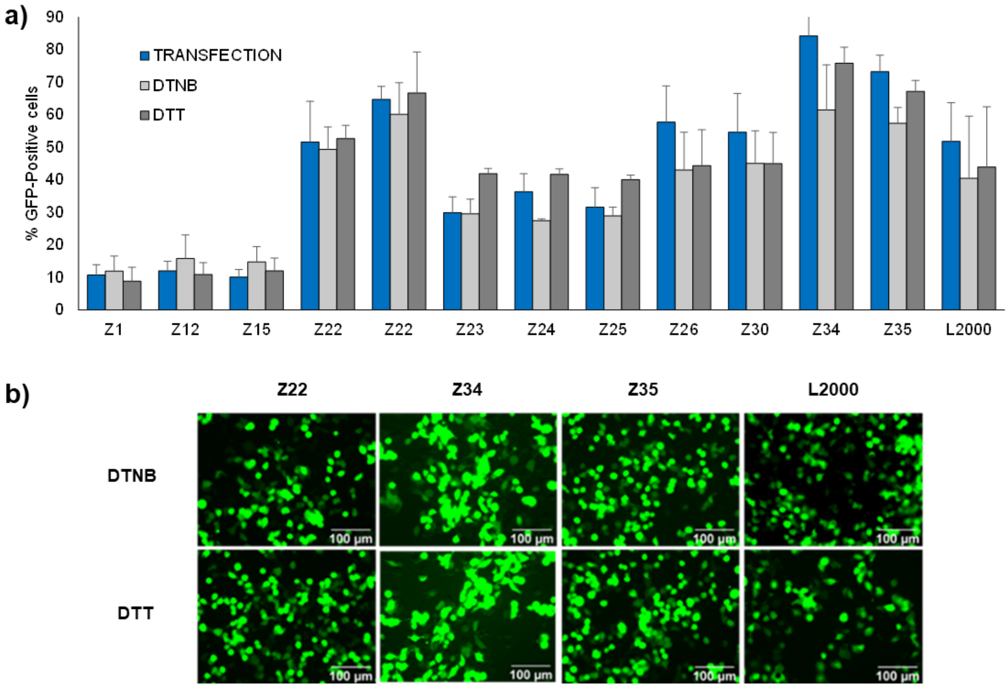

Figure S1 A) Transfection efficiency of peptide dendrimers/pDNA complexes on HEK293 cell in standard conditions (in blue), in the presence of 1.2 mM Protein disulfide isomerase inhibitor DTNB (in light grey), and 2 mM of Protein disulfide isomerase activator DTT (in dark grey). Cells are transfected with pDNA coding for CRISPR-Cas9 (CRISPRCas9/GFP, 250 ng). L2000 (2:1, v/w, L2000:DNA) is used as positive control.

Transfection efficiency is detected by FACS and expressed in percentage of transfected cells relative to the whole cell population ($1\cdot10^4$ events). Peptide dendrimers/pDNA complexes are formed at N/P 5 (1.75-2.4 µM/0.42 nM respectively). B) Fluorescent microscope images of HEK293 cells transfected in presence of DTT or DTNB. Pictures taken by Nikon Eclipse TS100 (20X objective) 48h after transfection. Scale bar 100µm.

2.4. Critical micellar concentration (CMC)

Nile red (Sigma Aldrich, Buchs, CH) was diluted in methanol at a concentration of 2 µM and 5 µL were added to each well of a TPP 96-well plate (Faust Laborbedarf AG, Schaffhausen) and dry under the fumehood air flow at room temperature for 1 h. Serial dilution of the peptide dendrimers was performed in 10 mM phosphate buffer (pH 5.0 or pH 7.4) starting from 0.625 mg/mL to 1 µg/mL and 50 µL was added to the plate containing the dried Nile red fluorophore (final concentration 0.2 µM). The plates were incubated for 2 h before measurement of fluorescence at λ_{ex}= 540 nm and λ_{em}= 615 nm on a Tecan Infinite M1000 Pro plate reader.

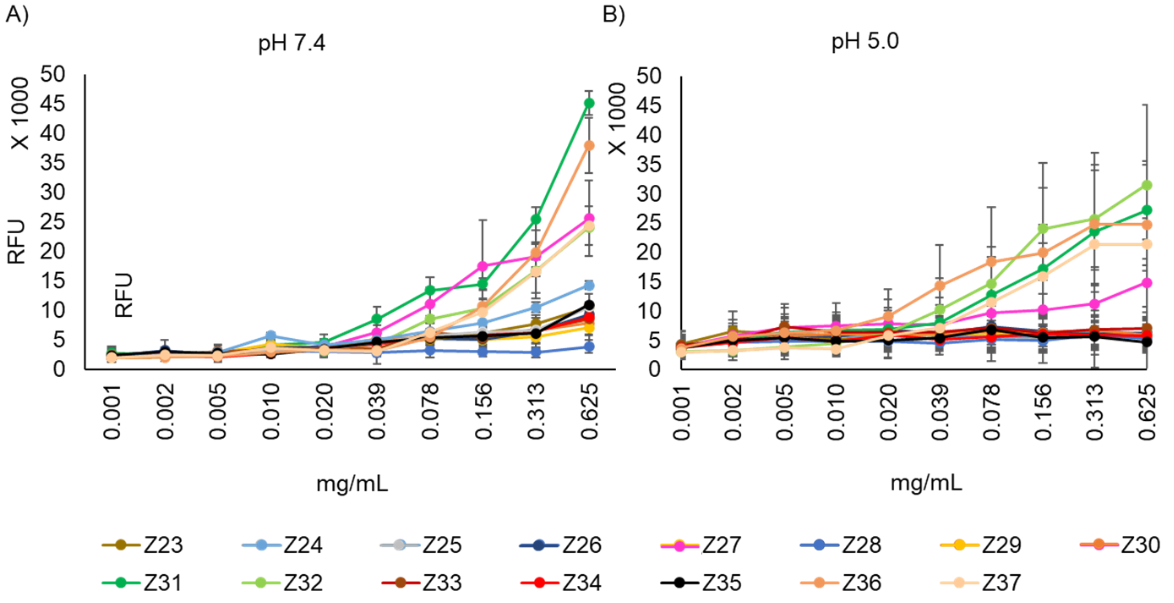

Figure S2. Critical aggregation concentration determination in phosphate buffer at A) pH 7.4 and B) pH 5.0, performed by serial dilution of compounds starting from 0.625 mg/mL to 1 µg/mL, followed by treatment of Nile red (final concentration of 0.2 µM). Fluorescence measured at λ_{ex} = 540 nm and λ_{em} = 615 nm. RFU = relative fluorescence unit.

2.5. Free pDNA assay by Quant-iT™ Picogreen®

The complexes were formed in OptiMEM by mixing pDNA (250 ng in 6.25 µL OptiMEM, 6.8 nM) with peptide dendrimers (N/P ratio of 5, 175-240 pmol, in 6.25 µL of OptiMEM, to achieve 27.9-38.4 µM) or L2000 (2:1, v/w, L2000:DNA, 0.5 µL from the 1 mg/mL commercial solution in 6.25 µL OptiMEM) for 30 min at room temperature (12.5 µL, concentration 3.4 nM

of pDNA and 14.0-19.2 μM of peptide dendrimer or 40 μg/mL of L2000). Then, the Quant-iT™ PicoGreen® dsDNA Reagent Kit (Thermo Fisher Scientific, Reinach, CH) was used following the manufacturer's protocol. Briefly, 1 μL of reagent was diluted in 200 μL of TE buffer and 195 μL added to the well of a TPP 96-well plate. Then, 5 μL of the complexes were added to the wells (200 μL, final concentration: 0.085 nM of pDNA and 0.35-0.48 μM of peptide dendrimer or 1 μg/mL of L2000) and fluorescence was measured at λ_{ex}= 480 nm and λ_{em}= 520 nm after 10 min on a Tecan Infinite M1000 Pro plate reader. The Quant-iT™ PicoGreen® signal from the complexes were normalized against a « pDNA alone » control to yield the percentage of the signal detected.

2.6. Internalization assay

HEK293 cells were transfected for 4 h with Cy3-labelled DNA prepared by using the Mirus DNA labelling kit (Mirus, Cambridge, UK) following the manufacturer's instructions. Each well was transfected with complexes following the standard procedure reported above. Following the 4h transfection, the cells were washed three times with heparin (200 μL/well; 2 mg/mL) in 1 h to remove the DNA complexes bound at the cell surface. Then, the cells were trypsinized, resuspended in full growth medium and transferred to flow cytometry tubes. The cellular uptake was detected by FACS analysis .

2.7. Confocal microscopy

Nunc Lab-Tek II 8-well chambered coverglass plates (Faust Laborbedarf AG, Schaffhausen) were pretreated with solution of poly-L-Lysine (Sigma Aldrich, Buchs, CH) for 1 h and dried at room temperature. Then, HEK293 cells were seeded and incubated overnight. Peptide dendrimer/pDNA complexes were formed as previously described by using Cy3-labelled pDNA. The complexes were added to the cells and incubated 4 h at 37°C in a humidified atmosphere in 5% carbon dioxide. Then the medium was removed, cells were washed with heparin in OptiMEM (2 mg/mL, 0.5 mL, 3 times) and washed twice with PBS. The cell membrane was labeled with CellMask Deep Red plasma membrane stain (Thermo Fisher Scientific, Reinach, CH), at 0.5X in full DMEM growth medium (0.25 μL in 0.5 mL / well), while the nuclei were labelled by 5 μM solution of Hoechst 33258 in DMEM for 30 minutes at 37°C. The cells were washed with PBS (1.0 mL/well, 3 times), FluoroBrite DMEM was added and images of live cells were taken on Leica SP8 confocal microscope.

2.8. pDNA transfection in presence of Bafilomycin A1

HEK293 cells were pretreated with Bafilomycin A1 (200 nM, Alfa Aesar, Karlsruhe, DE) in DMEM supplemented with 10% FCS for 1 h, followed by transfection, in 96-well TPP as described above. Peptide dendrimers/pDNA complexes were added to cells and incubated for 4 h at 37°C in a humidified atmosphere in 5% carbon dioxide. Then, complexes were removed and replaced with 100 µL of DMEM supplemented with 10% FCS containing 200 nM Bafilomycin A1 and incubated for 48 h. Transfection efficiency was assessed as described above after a total of 48 h.

2.9. Stability to proteinase K

Peptide dendrimers were prepared as 400 µM stock solutions in 10 mM Tris pH 8.0, 150 mM NaCl assay buffer with 4-hydroxybenzoic acid as internal standard (100 µg/mL). A proteinase K stock solution (80 µg/mL) was prepared in assay buffer. Proteolysis reactions were performed in a total volume of 500 µL and initiated upon addition of 250 µL (final concentration 200 µM) of the test peptide to 250 µL of Proteinase K (final concentration 40 µg/mL) at room temperature. At each time point, 50 µL of the reaction mixture was quenched in 100 µL of a 1:1 mixture acetonitrile/water + 1% TFA. Samples were injected and analyzed on an analytical C18 reverse-phase HPLC column. The amount of remaining peptide was quantified by integrating the peak area of the compound in the chromatogram and comparing it with the internal standard. The percentage remaining peptide was plotted against time.

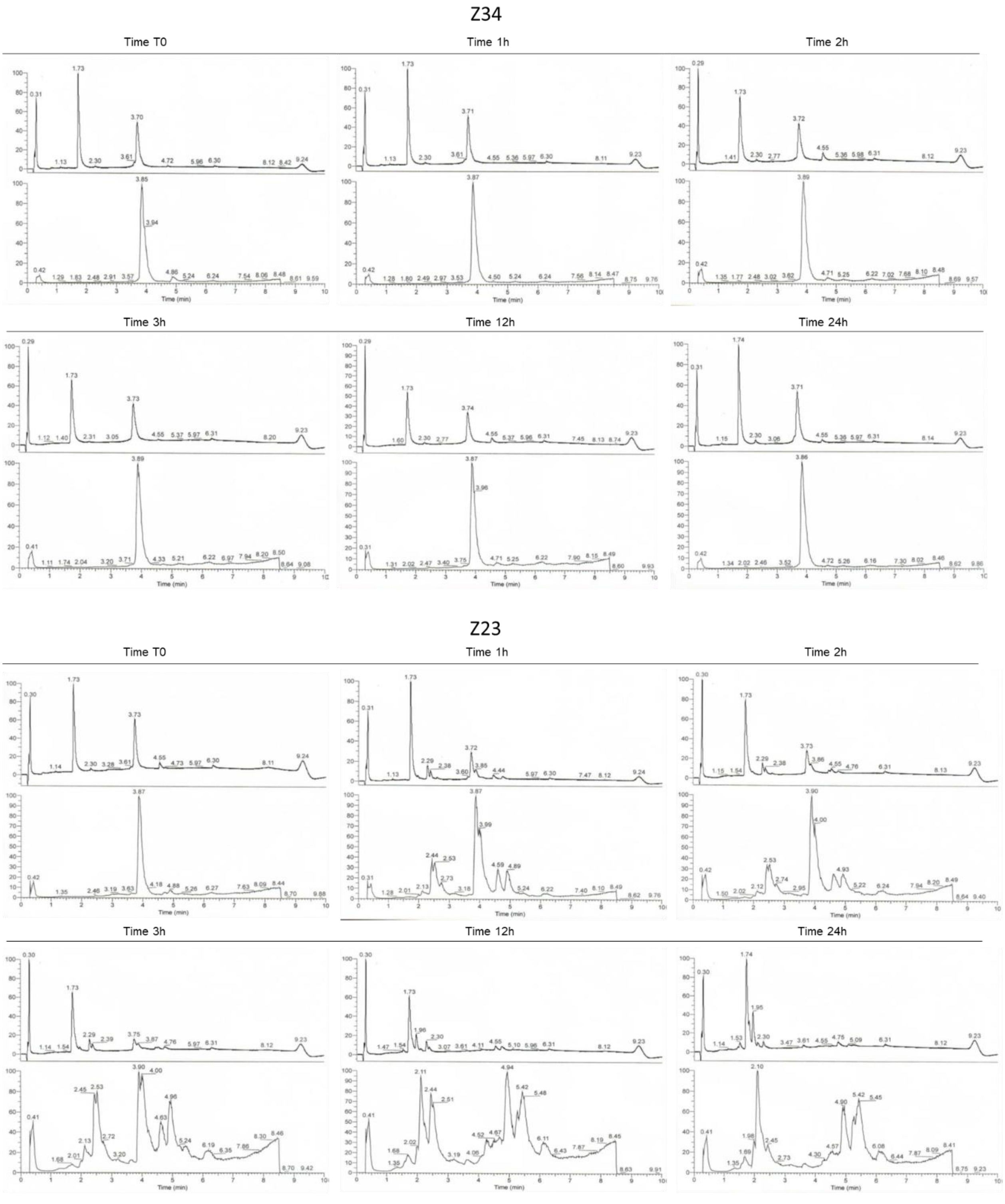

Figure S3. Primary data for stability of D-enantiomer **Z34** and its L-enantiomer **Z23** in the presence of proteinase K at different time points, determined by analytical HPLC/MS. Retention times: **Z34** and **Z23** at 3.73 min, internal standard (4-hydroxybenzoic acid) at 1.73 min in a 10 min run.

2.10. pDNA transfection in presence of protease inhibitors

HEK293 cells were treated with a protease inhibition cocktail containing serine, cysteine, aspartic proteases and aminopeptidase inhibitors (P1860-1Ml, Merck, components: Aprotinin, Bestatin, E-64, Leupeptin, Pepstatin A; Used following the recommendations: 1:200 diluition) in DMEM supplemented with 10% FCS for 1h before transfection, in 96-well TPP plates described above. Peptide dendrimers/pDNA complexes were added to cells and incubated for 4 h at 37°C in a humidified atmosphere in 5% carbon dioxide. Then, complexes were removed and replaced with 100 µL of DMEM supplemented with 10% FCS containing protease inhibition cocktail and incubated for 48 h. Transfection efficiency was assessed as described above after a total of 48 h.

2.11. pDNA transfection in presence of Dexamethasone

HEK293 cells were treated with Dexamethasone (5 µM) in DMEM supplemented with 10% FCS for 1 h before transfection, in 96-well TPP plates. Peptide dendrimers/pDNA complexes were added to cells by procedure described above and incubated for 4 h at 37°C in a humidified atmosphere in 5% carbon dioxide. Then, complexes were removed and replaced with 100 µL of DMEM supplemented with 10% FCS containing 5 µM of Dexamethasone and incubated for 48 h. Transfection efficiency was assessed as described above after a total of 48 h.

2.12. Measure of SpCas9 cleavage activity after pDNA* transfection by TIDE assay

sgRNA: 5'-CCTGGGTCCATCGCTCCGGCgttttagagctagaaatagcaagttaaaataaggctagtccgttat caacttgaaaaagtggcaccgagtcggtgc – 3'

HEK293 cells were seeded at $1{,}8\cdot10^5$ cells/well in 24 well plate, pretreated with solution of poly-L-Lysine for 1-2 h (until dryness). Transfection was performed under the standard conditions (N/P 5, peptide dendrimers/DNA complexes at 1.75 µM/ 0.42 nM respectively, L2000 at 5 µg/mL), with 4-fold final volume (400 µL/well) extension. Transfection solutions were added to cells for 4 h, followed by replacement with complete growth media and 48 h incubation at 37°C in a humidified atmosphere in 5% carbon dioxide. Cells were washed with PBS, trypsinized, (trypsin was inactivated by addition of full growth medium), centrifuged, resuspended in PBS and aliquoted in $\sim10^6$ cells. SpCas9 activity was measured following a published protocol for Tracking of Indels by Decomposition (TIDE)[3,4]. The extraction of genomic DNA was performed with ISOLATE II Genomic DNA Kit (Bioline) according to manufacturer's instructions. Amplification of the predicted cut site was performed with Q5 High-Fidelity DNA Polymerase (NEB) following manufacturer's instructions.The primers

TIDE_forward 5'- tgccaagaatccccgctatg -3' and TIDE_reverse 5'- cagatccaaggcctccactc -3' were designed using Primer-BLAST [5,6] and synthetised by Sigma-Aldrich. The amplification of a sharp single band was controlled by running the sample on agarose gel. The amplification product was sequenced by Sanger sequencing (Biochemistry Department, University of Cambridge, UK) from forward and reverse primers before analysis with the TIDE software. For each experiment, the samples were first analysed in batch using the automatic settings. If needed, the settings were then optimised for the batch in order to increase the reliability of the estimation. The TIDE software estimates the goodness of fit (R^2) between the sample and the model, a $R^2>0.9$ is recommended, therefore samples with $R^2<0.9$ – after settings optimisation – were removed from the analysis. The results were plotted an analysed using Prism (Version 9.0.0, GraphPad Software). Error bars display the standard error of the mean (SEM) calculated with the formula $SEM = \frac{\sigma}{\sqrt{N}}$ with σ being the standard deviation and N the sample size.

Table S2. Raw results from TIDE assay.

Experiment – forward			
TIDE settings	Alignment window		100 – 197 nt
	Decomposition window		222 – 685 nt
	Indels		10 bp
**Transfection agent**	Replicate	TIDE R^2	Overall efficiency (%)
**UTC**	1	CONTROL	0
**UTC**	2	0.99	4.1
**UTC**	3	0.86	16.2
**Z34**	1	0.98	13.3
**Z34**	2	0.98	12.2
**Z34**	3	0.99	10.3
**Z23**	1	0.93	13.4
**Z23**	2	0.99	3.3
**Z23**	3	0.99	5.1
**L2000**	1	0.99	21.3
**L2000**	2	0.99	20.4
**L2000**	3	0.99	20.8
**Experiment – reverse**			
TIDE settings	Alignment window		1 – 635 nt
	Decomposition window		660 – 685 nt
	Indels		10 bp
**Transfection agent**	Replicate	TIDE R^2	Overall efficiency (%)
**UTC**	1	CONTROL	0
**UTC**	2	1	3.4
**UTC**	3	1	0.6
**Z34**	1	1	10.9
**Z34**	2	1	8.5
**Z34**	3	1	11.6
**Z23**	1	1	3.1
**Z23**	2	0.99	3.6
**Z23**	3	1	3.2
**L2000**	1	0.99	19
**L2000**	2	0.99	19.5
**L2000**	3	0.99	18.4

03/04/21 Bern PCR with GUSB2 Ta=68°C

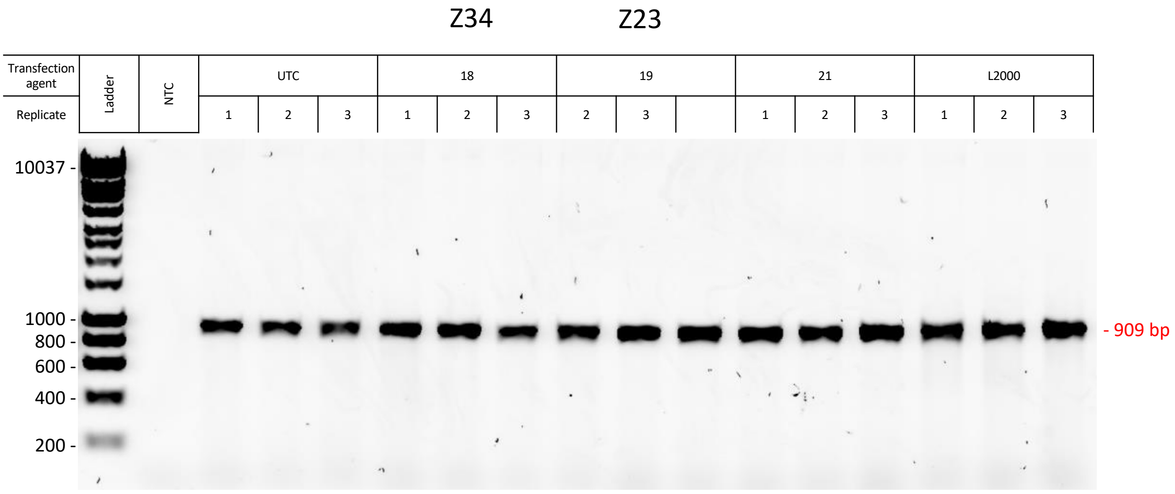

Figure S4. Amplification of genomic DNA. PCR amplification should give a single band at 909 bp.

3. References

(1) Zamolo, S. J.; Darbre, T.; Reymond, J.-L. Transfecting Tissue Models with CRISPR/Cas9 Plasmid DNA Using Peptide Dendrimers. *Chem. Commun.* **2020**, *56* (80), 11981–11984. https://doi.org/10.1039/D0CC04750C.

(2) Kwok, A.; Eggimann, G. A.; Reymond, J.-L.; Darbre, T.; Hollfelder, F. Peptide Dendrimer/Lipid Hybrid Systems Are Efficient DNA Transfection Reagents: Structure–Activity Relationships Highlight the Role of Charge Distribution Across Dendrimer Generations. *ACS Nano* **2013**, *7* (5), 4668–4682. https://doi.org/10.1021/nn400343z.

(3) Brinkman, E. K.; Chen, T.; Amendola, M.; van Steensel, B. Easy Quantitative Assessment of Genome Editing by Sequence Trace Decomposition. *Nucleic Acids Research* **2014**, *42* (22), e168–e168. https://doi.org/10.1093/nar/gku936.

(4) Brinkman, E. K.; van Steensel, B. Rapid Quantitative Evaluation of CRISPR Genome Editing by TIDE and TIDER. In *Methods in Molecular Biology*; Humana Press Inc., 2019; Vol. 1961, pp 29–44. https://doi.org/10.1007/978-1-4939-9170-9_3.

(5) Ye, J.; Coulouris, G.; Zaretskaya, I.; Cutcutache, I.; Rozen, S.; Madden, T. L. Primer-BLAST: A Tool to Design Target-Specific Primers for Polymerase Chain Reaction. *BMC bioinformatics* **2012**, *13* (1), 134. https://doi.org/10.1186/1471-2105-13-134.

(6) Kõressaar, T.; Lepamets, M.; Kaplinski, L.; Raime, K.; Andreson, R.; Remm, M. Primer3_masker: Integrating Masking of Template Sequence with Primer Design Software. *Bioinformatics* **2018**, *34* (11), 1937–1938. https://doi.org/10.1093/bioinformatics/bty036.
